# The Evolving Landscape of Clinical Aging Clocks: From Epigenetic to Multi‐Omics Integration

**DOI:** 10.1111/acel.70579

**Published:** 2026-06-16

**Authors:** Liying Liu, Yuanyuan Lai, Chunhui Tian, Yufei Huang, Jianheng Hao, Yuemeng Zhao, Dan Chen, Tianyu Wu, Daqian Zhou, Xiaoyan Zheng, Han Yang, Zheng Yu, Nihong Li, Jie Yang

**Affiliations:** ^1^ Acupuncture and Tuina School Chengdu University of Traditional Chinese Medicine Chengdu China; ^2^ Department of Clinical Medicine Shanxi University of Medicine Lvliang China; ^3^ Department of Acupuncture and Moxibustion Shenzhen Bao'an District Hospital of Traditional Chinese Medicine Shenzhen Guangdong China; ^4^ Division of Internal Medicine, Institute of Integrated Traditional Chinese and Western Medicine, West China Hospital Sichuan University Chengdu China; ^5^ School of Intelligent Medicine Chengdu University of Traditional Chinese Medicine Chengdu China; ^6^ Reproductive Center The Fifth People's Hospital of Chengdu University of Traditional Chinese Medicine Chengdu China; ^7^ Department of Traditional Chinese Medicine, West China Second University Hospital Sichuan University, West China Women's and Children's Hospital Chengdu China

**Keywords:** aging clocks, deep learning, ensemble learning, epigenetic, multi‐omics

## Abstract

Aging clocks are tools that quantify biological aging through the integration of multi‐omics data, encompassing epigenetic, transcriptomic, proteomic, metabolic, and microbial information, together with functional biomarkers. These tools show significant potential for use in preventive medicine, early detection of chronic conditions, and monitoring the effectiveness of interventions designed to enhance population health. The advancement of artificial intelligence has facilitated the widespread adoption of ensemble learning and deep learning techniques in constructing aging clocks. Such methods are capable of efficiently synthesizing and analyzing high‐dimensional, multi‐modal biological data, thereby uncovering deeper insights and promoting a transition from epigenetic‐based aging prediction to the development of multi‐omics and multi‐modal aging clocks. Aging clocks built on large‐scale data and artificial intelligence have demonstrated notable progress in terms of accuracy, interpretability, and generalizability. Consequently, they provide a substantive foundation for understanding mechanisms of aging and contribute meaningful guidance for clinical practices aimed at delaying age‐related diseases and fostering healthy aging.

## Graphical abstract

Multi‐omics aging clocks capture biological heterogeneity beyond single‐omics models, improving risk stratification. Key challenges include unknown biological meaning, timescale mismatches, and validation gaps. Future clocks must distinguish pathological damage from adaptive remodeling—damage accumulation is the most actionable target for anti‐aging interventions.

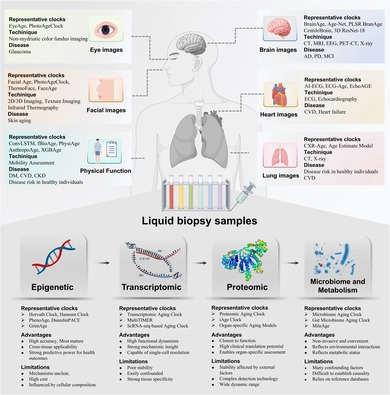

**Abbreviations**
5mC: 5‐methylcytosine
ABC: aging biomarker consortium
AD: Alzheimer's disease
AGE: advanced glycation end product
AI: artificial intelligence
ATAC‐seq: assay for transposase‐accessible chromatin using sequencing
Cf‐DNA: cell‐free DNA
CHARLS: China Health and Retirement Longitudinal Study
CNNs: convolutional neural networks
CpG: cytosine‐phosphate‐guanine
CT: computed tomography
DMN: default mode network
DNNs: deep neural networks
DunedinPACE: Dunedin Pace of Aging Calculated from the Epigenome
ECG: electrocardiograms
EEAA: extrinsic epigenetic age acceleration
EEG: electroencephalogram
FBioAge: functional biological age
GPR: Gaussian process regression
GPT: generative pre‐trained transformer
GTEx: genotype‐tissue expression
HAM: healthy aging metabolomic
IAge: inflammatory aging clock
IEAA: intrinsic epigenetic age acceleration
IL‐6: interleukin‐6
LSTMs: long short‐term memories
MAE: mean absolute error
MCI: mild cognitive impairment
ML: machine learning
MRI: magnetic resonance imaging
MSE: mean squared error
PBMCs: peripheral blood mononuclear cells
PCA: principal component analysis
PCA: principal component analysis
PD: Parkinson's disease
Qnorm: quantile normalization
REs: repetitive elements
RNNs: recurrent neural networks
SASP: senescence‐associated secretory phenotype
ScRNA‐seq: single‐cell RNA sequencing
SEA: sperm epigenetic age
SenCID: senescent cell identification
Senoproteins: senescence‐associated proteins
SHAP: SHapley Additive exPlanations
SVMs: support vector machines

## 1 Introduction

The process of aging is characterized by a progressive decline in physiological function and an increased risk of age‐related diseases, underscoring the need to objectively quantify biological aging and reflect systemic changes at molecular and functional levels (Bao et al. 2023; Moqri et al. 2024; Wu et al. 2025). Aging clocks are broadly defined as quantitative biomarkers of aging and can be constructed using diverse computational strategies. Many are trained to predict chronological age from age‐informative molecular features, with the difference or residual between predicted and chronological age subsequently interpreted as a measure of biological age acceleration (Skinner et al. 2025). Others are constructed to estimate biological age or aging‐related risk more directly by integrating molecular, physiological, or clinical features associated with functional decline, morbidity, or mortality, and some are further designed to capture longitudinal change and thereby quantify the pace of aging (Bafei and Shen 2023). Despite these differences in implementation and target outcome, all aim to capture aging‐related variation beyond chronological time. Some clocks are intended to reflect organism‐level aging, whereas others are trained in specific tissues or cell types and therefore capture tissue‐ or cell‐specific aging patterns relative to chronological age (Sehgal 2025). Thus, clocks across different omics layers may differ not only in computational objective, but also in whether they represent systemic aging or context‐dependent aging within a particular biological compartment.

A landmark contribution came from Steve Horvath, who developed the first multi‐tissue epigenetic clock based on DNA methylation in 2013. Constructed from 7844 public microarray samples across 51 healthy tissues and cell types, the Horvath Clock used 353 Cytosine‐phosphate‐Guanine (CpG) sites to accurately predict age across diverse biological sources (Horvath 2013). Although it has been validated in numerous independent studies (Horvath and Raj 2018; Zhang, Reynolds, et al. 2024), its performance varies across certain contexts such as blood and hormonally sensitive tissues (Olesen et al. 2018). Horvath's work significantly inspired further study, leading to the development of aging clocks with multidimensional and high‐resolution approaches (Teschendorff and Horvath 2025). Clinical aging clocks utilize biomolecular data from invasive or non‐invasive human samples to quantify biological age and assess health risks, thereby supporting early disease intervention and personalized health strategies. Nevertheless, their development faces practical constraints such as limited bio‐sample availability (Qi and Teschendorff 2022) and computational challenges (Teschendorff and Relton 2018). Recent advances in multi‐omics (Mavromatis et al. 2023) and artificial intelligence (AI) (Qiu et al. 2023; Kalyakulina et al. 2024) are addressing these limitations, enabling the construction of more accurate and multidimensional aging models.

Firstly, aging clocks have evolved from relying solely on DNA methylation to integrating multi‐omics data (Jylhävä et al. 2017), spanning epigenomic (Horvath 2013; McCrory et al. 2020), transcriptomic (Meyer and Schumacher 2021; Lu, Brbić, et al. 2023), proteomic (Johnson et al. 2020), metabolomic (Jia et al. 2024), and microbiome layers (Wilmanski et al. 2022) to capture aging across biological scales from individuals to organs and cells. These integrated models improve assessment of population‐level aging and disease‐specific risk (Moqri et al. 2023). Although easily accessible biospecimens (e.g., blood) are widely used, they offer limited insight into organ‐specific aging (Kusters and Horvath 2025). Recent studies have combined transcriptomic data from Genotype‐Tissue Expression (GTEx) with proteomic data from UK Biobank to identify organ‐specific aging markers and build organ‐level clocks, improving risk prediction for diseases in specific organs (Lehallier et al. 2019; Goeminne et al. 2025). Furthermore, single‐cell omics technologies have unveiled cell‐type‐specific aging signatures, leading to the development of cellular‐level models such as the Microglia scRNA‐seq Aging Clock (Stanley et al. 2025) and Senescent Cell Identification (SenCID) (Tao et al. 2024). These tools help elucidate cell‐type‐specific genetic regulatory dynamics and capture key cellular changes during aging. These advances facilitate a deeper understanding of aging dynamics across cellular populations.

Secondly, AI algorithms are crucial for integrating high‐dimensional data (Hong et al. 2024) and identifying complex aging patterns (Kale et al. 2024). While penalized linear regression models remain popular for their interpretability and simplicity (Meng et al. 2024), the nonlinear nature of aging (Moskalev et al. 2014; Shen et al. 2024; Ding et al. 2025) has motivated the adoption of more advanced AI techniques. Ensemble methods like XGBoost and LightGBM improve predictive accuracy and robustness by combining multiple weak learners (Shokhirev et al. 2024; Khodasevich et al. 2025). For instance, CheekAge leveraged the XGBoost algorithm to capture nonlinear relationships between lifestyle factors and epigenetic age (Shokhirev et al. 2024). Deep neural networks (DNNs) have demonstrated exceptional capability in modeling complex mappings between medical imaging and omics data (Shokhirev et al. 2024). The DeepMAge model developed by Galkin et al. (2021) employs a DNN architecture that uses backpropagation to adjust weights and establish a continuous nonlinear function linking methylation β‐values to biological age, thereby achieving effective nonlinear feature mapping from raw DNA methylation data to biological age estimates (Galkin et al. 2021, 2022). More recently, transformer‐based models have been applied to multi‐omics integration, using self‐attention mechanisms to capture long‐range dependencies and identify disease‐relevant pathways (Urban et al. 2023).

This review focuses on human aging research, systematically summarizing recent advances based on multidimensional biomarkers, including functional measures, imaging data, and biofluids. This review aims to offer key insights for developing clinically useful aging assessment tools and improving personalized health intervention strategies, thereby helping to bridge the gap between aging research and clinical practice.

### 1.1 Clinical Biosamples and Aging Clock Classification

A recent review identified 12 hallmarks of aging, including genomic instability, telomere shortening, epigenetic alterations, loss of proteostasis, and chronic inflammation, among others (López‐Otín et al. 2023). However, clinical practice remains constrained by the limited predictive capability of any single indicator, underscoring the need for integrative models such as aging clocks. Clinical aging clocks can be broadly categorized into two types: molecular‐level aging clocks and function‐oriented aging clocks. Molecular‐level aging clocks represent models derived from high‐dimensional omics data, including epigenomic, transcriptomic, proteomic, metabolomic, and microbiome profiles. These clocks commonly utilize both invasive and non‐invasive biospecimens. In contrast, function‐oriented aging clocks represent models based on physiological and functional assessments, such as imaging metrics and mobility tests, and primarily rely on non‐invasive samples.

**FIGURE 1:**
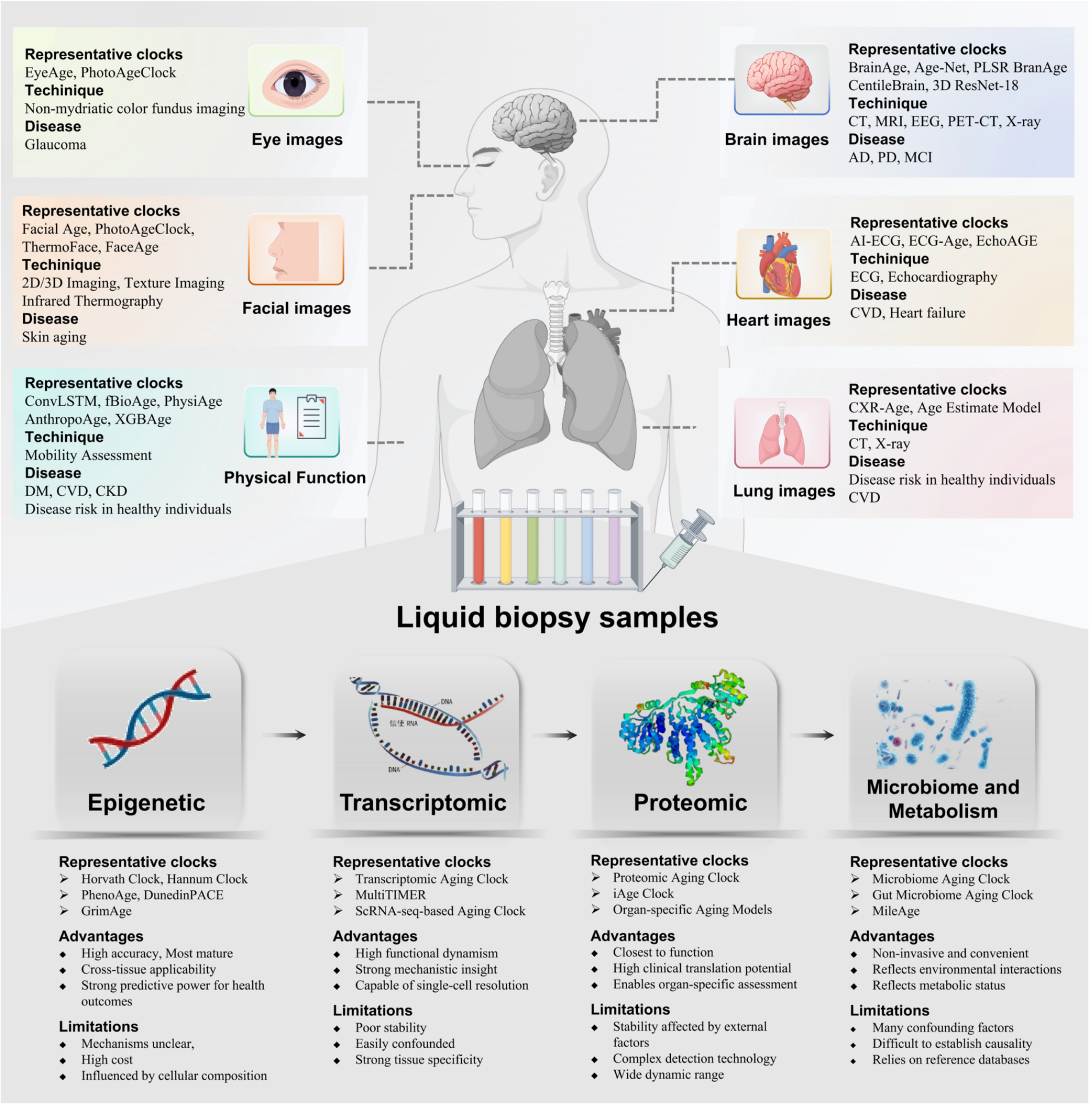
Overview diagram of aging clocks. AD, Alzheimer's disease; CKD, chronic kidney disease; CVD, cardiovascular disease; DM, diabetes mellitus; MCI, mild cognitive impairment; PD, Parkinson's disease.

In the development of clinical aging clocks, researchers utilize a wide range of biological samples and functional data, which can broadly be categorized into invasive and non‐invasive types. Invasive samples, valued for their rich molecular information, serve as a fundamental resource for constructing molecular‐level aging clocks. Blood samples are the most extensively used material in aging clock research, valued for their low invasiveness and molecular richness, which support the development of clocks that capture diverse aspects of the aging process. Specifically, whole blood (Hannum et al. 2013; Snir et al. 2016; Paparazzo et al. 2022) is used for multi‐omics studies such as whole‐genome sequencing (Hannum et al. 2013; Schmunk et al. 2025) and methylation profilin (Martínez‐Enguita et al. 2024; Zhang, Reynolds, et al. 2024; Zheng et al. 2024); Plasma (Lehallier et al. 2020; Liang et al. 2020; Goeminne et al. 2025) or serum (van den Akker et al. 2020; Hamsanathan et al. 2024; van Holstein et al. 2024) is used for proteomic mass spectrometryand cell‐free DNA (cf‐DNA) sequencing; While peripheral blood mononuclear cells (PBMCs) are commonly used for single‐cell RNA sequencing (scRNA‐seq) (Zhu, Chen, et al. 2023; Stanley et al. 2025) and epigenomic analyses such as assay for transposase‐accessible chromatin using sequencing (ATAC‐seq) (Ranzoni et al. 2021; Grandi et al. 2022; Morandini et al. 2024). Additionally, stool samples are analyzed via metagenomic sequencing (Galkin et al. 2020; Chen et al. 2022) or 16S rRNA amplicon sequencing (Odamaki et al. 2016) to investigate the relationship between gut microbiota and aging. Oral swabs and saliva samples leverage cfDNA derived from oral epithelial cells for epigenetic profiling (Zhang et al. 2019; McEwen et al. 2020; Graw et al. 2021; Chen et al. 2022; Dec et al. 2023; Shi et al. 2023; Shokhirev et al. 2024; Shtumpf et al. 2024). Cerebrospinal fluid offers a unique source of biomarkers for central nervous system aging (Hwangbo et al. 2022; Heming et al. 2025). Non‐invasive samples are primarily used to establish function‐oriented aging assessment models, encompassing various imaging and physiological functional data. For example, facial images are used with deep learning algorithms to evaluate skin aging (Wan et al. 2018; Wang et al. 2023; Yu, Zhou, et al. 2024; Bontempi et al. 2025); Fundus imaging serves as a systemic aging assessment tool, predicting mortality risk, indicating multi‐organ aging mechanisms, and revealing synergistic effects between multiple pathological factors and ocular aging (Bobrov et al. 2018; Shokhirev and Johnson 2021; Ahadi et al. 2023; Zhu, Chen, et al. 2023). Electrocardiograms (ECG) (Shelly et al. 2023; Evans et al. 2025; Liu, Kuo, et al. 2025) and echocardiography (Kobelyatskaya, Guvatova, et al. 2024) help infer cardiovascular functional age. X‐ray imaging (Raghu et al. 2021), computed tomography (CT) (Kerber et al. 2023) and magnetic resonance imaging (MRI) (Monti et al. 2020; Hu et al. 2023; Guan et al. 2024; Riccardi et al. 2025) assess structural age of organs such as bones and the brain. Electroencephalogram (EEG) (Fingelkurts and Fingelkurts 2023) captures neuroelectro physiological aging, and physical performance tests (such as gait speed, balance ability) evaluate overall functional status (Rahman and Adjeroh 2019; Sternäng et al. 2019; Janssens et al. 2023; Shim et al. 2024). Based on these sample types, invasive samples mainly support the development of molecular‐level aging clocks, while non‐invasive samples form the basis for the function‐oriented aging clock (see Figure 1).

## 2 Molecular‐Level Aging Clock

### 2.1 Epigenetic Clock

The epigenetic clock is currently the most advanced and widely used aging model (Teschendorff and Horvath 2025). Specifically, DNA methylation involves the covalent attachment of a methyl group to cytosine, forming 5‐methylcytosine (5mC) (Day et al. 2013), which mainly occurs at CpG dinucleotide regions. During aging, CpG sites in promoter regions often become hypermethylated, while those in other areas may become hypomethylated (Saul and Kosinsky 2021), forming the basis of the epigenetic clock (Li et al. 2022). To date, three generations of epigenetic clock models have been developed.

The first‐generation epigenetic clocks primarily modeled chronological age (Declerck and Vanden Berghe 2018) as a function of DNA methylation levels (Griffin et al. 2024). The Horvath Clock, developed using 7844 microarray samples from 51 healthy tissues and cell types, was the first multi‐tissue age predictor based on 353 CpG sites (Horvath 2013). It has been validated across hundreds of independent datasets (Horvath and Raj 2018; Zhang, Reynolds, et al. 2024), although its prediction precision varies in hormonally sensitive tissues and blood (Olesen et al. 2018). In the same year, Hannum et al. (2013) developed another highly accurate epigenetic clock based on methylation levels at 71 CpG sites in whole blood, providing a blood‐based estimator of chronological age.

Given the limitations of early blood‐based clocks, more targeted epigenetic clocks have been developed for specific research areas. First, sex differences affect the accuracy of these clocks, with males typically exhibiting higher epigenetic age than females in blood samples (Horvath et al. 2016; Phyo et al. 2024). Vidaki et al. (2021) developed a Y‐chromosome‐based clock (Y‐CpG Age) to correct for sex bias, offering higher stability for male aging research and forensic estimation. Similarly, Pilsner et al. (2022) developed the Sperm Epigenetic Age (SEA) Clock based on sperm DNA methylation using ensemble machine learning algorithms. Sawant later linked the SEA Clock to semen parameters, finding associations with sperm head morphology abnormalities, offering new molecular indicators for assessing male fertility (Sawant et al. 2024). Researchers have also developed population‐specific clocks for different life stages. The PedBE Clock, based on DNA methylation profiles from buccal epithelial cells in children, showed strong correlations with chronological age (McEwen et al. 2020). For very young populations, clocks based on umbilical cord blood (Knight et al. 2016; Monasso et al. 2021) and placental tissue (Mayne et al. 2017; Lee et al. 2019) have been created to estimate gestational age, with findings linking placental aging to preeclampsia and prenatal depression. Building on this, the NEOAge was developed using buccal mucosa cells to assess neonatal aging in preterm infants while also estimating gestational age (Graw et al. 2021). For the oldest old, Dec et al. (2023) developed the Centenarian Clock to detect different aging patterns across age groups based on whole‐genome methylation profiling.

Compared with first‐generation epigenetic clocks, second‐generation clocks aim not only to estimate biological age but also to better reflect health status and disease risk (Belsky et al. 2020; Liang et al. 2024). Among these, PhenoAge (Levine et al. 2018), GrimAge (Lu, Quach, et al. 2019), and Dunedin Pace of Aging Calculated from the Epigenome (DunedinPACE) (Belsky et al. 2022) are prominent examples. Developed from the U.S. NHANES III dataset (Levine et al. 2018), PhenoAge uses nine clinical biomarkers linked to DNA methylation patterns to identify 513 CpG sites. It predicts age‐related diseases, functional decline, comorbidities, and all‐cause mortality, outperforming first‐generation clocks in predicting 10‐ and 20‐year mortality risk (Li et al. 2022). Liu et al. (2024) showed that vitamin D and exercise synergistically slowed biological aging as measured by PhenoAge. Building on PhenoAge, GrimAge was developed to predict lifespan and mortality more accurately (Lu, Quach, et al. 2019). Its updated version, GrimAge2 (Lu et al. 2022), incorporates inflammatory and metabolic biomarkers, broadening its use to metabolic syndrome monitoring and organ fat assessment—though it remains less responsive to short‐term health changes (Unnikrishnan et al. 2019). DunedinPoAm and DunedinPACE were developed from long‐term data on 954 individuals aged 26 to 38, monitoring 18 biomarkers over time, strengthening the link between biological aging rates and health outcomes (Belsky et al. 2022).

Age‐related DNA methylation changes arise from both directed biological processes and stochastic variation shaped by genetic, environmental, and lifestyle factors. This stochasticity follows an age‐related constraint described as quasi‐randomness (Minteer et al. 2023; Meyer and Schumacher 2024; Tarkhov et al. 2024; Tong et al. 2024). Tong et al. (2024) showed that synthetic clocks reproduced 66%–75% of the predictive accuracy of the Horvath clock, 90% of that of ZhangAge, but only 63% of PhenoAge, suggesting that clocks trained on chronological age rely more heavily on stochastic drift, whereas second‐generation clocks capture a larger non‐random component related to biological deterioration. At the same time, large methylation datasets and cross‐platform measurements can introduce technical noise, reducing clock reliability in longitudinal studies (Graw et al. 2021; Cao et al. 2022). To address this issue, Higgins‐Chen et al. (2022) proposed a principal component analysis (PCA)‐based approach that improves clock robustness by separating stable shared signal from technical variation. In older U.S. adults, PCA adjustment improved PhenoAge performance, left GrimAge largely stable, and reduced the predictive ability of DunedinPACE, indicating that denoising effects depend on model design (Faul et al. 2023). This is consistent with DunedinPACE's distinct training framework, which captures the pace of aging rather than cross‐sectional age (Belsky et al. 2022). Beyond stochastic modeling, denoising, and longitudinal training, causality‐enriched frameworks such as CausAge, AdaptAge, and DamAge further separate adaptive methylation changes from damaging ones (Ying et al. 2024). Together, these advances suggest that future epigenetic clocks should integrate stochastic modeling, PCA‐based denoising, longitudinal modeling, and causal decomposition to distinguish time‐dependent epigenetic drift from damage‐related signals that more directly drive functional decline, morbidity, and mortality (Tables 1 and 2).

**TABLE 1:** The summary of omics‐based aging clocks.

Generation	Name	Year	Sample size of training/test set	Ethnicity	Primary features	Internal training tissue type	External validation sample size/ethnicity/tissue type	Platform	Outcomes
The First Generation Epigenetic Clock	Horvath Clock (Horvath 2013)	2013	7844	NA	353 CpGs	Multiple tissues (51 types)	NA	Illumina 27 K, 450 K	① Predict chronological age and calculate age acceleration.
	Hannum Clock (Hannum et al. 2013)	2013	656/687	Multiracial	71 CpGs	Whole blood	NA	Illumina 450 K	① Predict chronological age and calculate age acceleration.
	WeidnerAge (Weidner et al. 2014)	2014	82/NA	Multiracial	3 CpGs	Whole blood	NA	Illumina 27 K	① Predict chronological age; ② Correlation between biological age and clinical and lifestyle parameters; ③ iPSCs can reverse age‐related methylation changes.
	VidalBraloAge (Vidal‐Bralo et al. 2016)	2016	390/427	Multiracial/NA	8 CpGs	Whole blood	427/European/Whole blood	Illumina 27 K, 450 K	① Predict chronological age.
	DNAm GA (Knight et al. 2016)	2016	207/1227	Multiracial	148 CpGs	Umbilical cord blood, blood spot	NA	llIumina 27 K, 450 K	① Predict gestational age and calculate gestational age acceleration; ② Correlation between gestational age acceleration and birthweight as well as maternal medicaid status.
	ZhangAge (Zhang et al. 2019)	2019	13,661/stratified cross‐validation	Multiracial	514 CpGs	Whole blood, saliva	1342/NA/Whole blood, endometrium, brain, breast, liver, fat, muscle, saliva	Illumina 450 K, EPIC	① Predict chronological age and calculate age acceleration; ② Correlation between age acceleration and mortality rate.
	Placental Clock (Lee et al. 2019)	2019	RPC: 1102/187; CPC: 963/187; Refined RPC: 733/187	NA	RPC: 558 CpGs; CPC: 546 CpGs; Refined RPC: 395 CpGs	Placenta	187/NA/Placental tissue, umbilical cord blood, skin, blood	Illumina 450 K, EPIC	① Predict gestational age and calculate gestational age acceleration.
	epiTOC2 (Teschendorff 2020)	2020	656/2586	Multiracial/NA	163 CpGs	Multiple tissues (19+ types)	7005/NA/Multiple tissues (56+ types)	Illumina 450 K	① Predict the rate of aging; ② Association of the rate of aging with the risk of chronic inflammation and cancer.
	PedBE Clock (McEwen et al. 2020)	2020	1032/689	Multiracial/NA	94 CpGs	BEC of children	1057/NA/BEC, saliva, blood	Illumina 450 K, EPIC	① Predict chronological age; ② Related to autism spectrum disorder.
	MethylNet (Levy et al. 2020)	2020	503/144	NA	200,000 to 300,000 CpGs	Whole blood, cancer tissues (32 types)	1129/NA/Whole blood, breast tumor tissue	Illumina 450 K, EPIC	① Predict chronological age; ② Related to immune cell proportions, pan‐cancer subtypes, and smoking status.
	DNAge Clock (Monseur et al. 2020)	2020	39	Multiracial	500+ CpGs	Whole blood	NA	Illumina HiSeq 1500	① Predict chronologicall age and calculate age acceleration; ② Correlation between age acceleration and ovarian reserve function.
	Robust Epigenetic Model (Montesanto et al. 2020)	2020	247/83	European	8 CpGs	Whole blood	NA	Sequenom MassARRAY	① Predict chronological age.
	Bohlin Clock (Monasso et al. 2021)	2021	1068/685	European	96 CpGs	Umbilical cord blood	NA	Illumina 450 K	① Predict gestational age.
	NEOAge (Graw et al. 2021)	2021	542/48	Multiracial	303–522 CpGs	BEC	48/NA/saliva	Illumina 450 K, EPIC	① Predict chronological age; ② Association of chronological age with complications during the neonatal period and long‐term diseases.
	Y‐CpG Age (Vidaki et al. 2021)	2021	758/127	European and American	75 Y‐CpGs	Whole blood	127/NA/Peripheral leukocytes	Illumina 450 K	① Predict male chronological age; ② Correlation between chronological age and male reproductive function.
	Paparazzo Clock (Paparazzo et al. 2022)	2022	194/NA	European	10 CpGs	Whole blood	NA	Illumina 450 K, EPIC	① Predict chronological age; ② Correlation chronological age and lipid metabolism (ELOVL2) as well as immune function (sjTREC).
	Pan‐tissue Methylation Aging Clock (Vijayakumar and Cho 2022)	2022	3114/1557	NA	6761 CpGs	Multiple tissues (20 types)	305/NA/Colon, Heart, Lung, Glial cells, Neurons	Illumina 450 K, EPIC	① Predict pan‐tissue chronological age and calculate age acceleration.
	SEA Clock (Pilsner et al. 2022)	2022	379/10 FCV	Multiracial	SEA_CpG_: 120CpGs, SEA_DMR_: 117CpGs	Semen	173/NA/Semen	Illumina EPIC	① Predict the chronological age and age acceleration of sperm; ② Correlation between age acceleration and sperm function indicators.
The First Generation Epigenetic Clock	Centenarian Clock (Dec et al. 2023)	2023	7039/Split randomly into 20 folds	Multiracial	33,495 CpGs	Blood, saliva, buccal mucosal cells	7227/Multiracial/Urine	Illumina 450 K, EPIC	① Predict chronological age of centenarians and calculate age acceleration; ② Age acceleration links clinical biomarkers and all‐cause mortality.
	GP‐age (Varshavsky et al. 2023)	2023	7860/3385	Multiracial	30 CpGs	Whole blood	675/European and American/Whole blood	Illumina 450 K, EPIC	① Predict chronological age and calculate age acceleration.
	StocAge (Tong et al. 2024)	2024	NA	NA	StocH: 353 CpGs; StocZ: 514 CpGs; StocP: 513 CpGs; StocM: 163 CpGs	NA	23,344/Multiracial/Blood, lungs, breasts, stomach, esophagus, colon	Illumina 450 K, EPIC	① Predict chronological age and calculate age acceleration; ② Quantify the contribution of random components to epigenetic aging
	PerSEClock (Zhao et al. 2024)	2024	8577/1059	NA	24,516 CpGs	Multiple tissues (8+ types)	3069/NA/Multiple tissues (8+ types)	Illumina 27 K, 450 K, EPIC	① Predict chronological age and calculate age acceleration.
	TWBAge; TWBhAge (Huang et al. 2025)	2025	TWBAge: 1235/493; TWBhAge: 692/277	Asian	TWBAge: 325 CpGs; TWBhAge: 179CpGs	Serum	NA	Illumina EPIC	① Predict chronological age and calculate age acceleration; ② Correlation between age acceleration and long‐term air pollution exposure.
The Second‐generation Epigenetic Clock	epiTOC (Yang et al. 2016)	2016	656/335	European	385 CpGs	Whole blood; normal colon, adjacent tissues (kidney, liver, lung)	6312/NA/Multiple tissues (31 types)	Illumina 450 K	① Predict chronological age; ② Correlation between epiTOC score and smoking pack‐years and cancer risk.
	EEAA; IEAA (Quach et al. 2017)	2017	4173	Multiracial	EEAA: 71 CpGs; IEAA: 353 CpGs	Whole blood	402/European/Whole blood	Illumina 450 K	① Predict chronological age and calculate age acceleration; ② Association of age acceleration and the risk of aging‐related diseases, diet, alcohol, education, BMI, as well as metabolism.
	SkinBloodAge (Horvath and Raj 2018)	2018	896/1622	Multiracial	391 CpGs	Multiple tissues (8+ types)	WHI Cohort: 3700/NA/Whole blood; Framingham Cohort: 905/NA/Whole blood; Jackson Cohort: 1639/American/Whole blood	Illumina 450 K, EPIC	① Predict chronological age and calculate age acceleration; ② Association of biological age and the all‐cause mortality rate, lifestyle, physical indicators.
	PhenoAge (Levine et al. 2018)	2018	10,382/6209	European and American/Multiracial	513 CpGs	Whole blood	9164/Multiracial/ Multiple tissues (12+ types)	Illumina 450 K, EPIC	① Predict biological age and calculate age acceleration; ② Association of biological age with the risk of mortality, CVD, diabetes, lung cancer, and AD.
	GrimAge (Lu, Quach, et al. 2019)	2019	1731/625	Multiracial	1030 CpGs	Whole blood	7375/Multiracial/Whole blood	Illumina 450 K, EPIC	① Predict biological age and calculate age acceleration; ② Association of age acceleration and all‐cause mortality, diseases, risk of menopause age, as well as lifestyles.
	DNAmTL (Lu, Seeboth, et al. 2019)	2019	2256/1078	Multiracial	140 CpGs	Multiple tissues (7 types)	9345/Multiracial/ Multiple tissues (4 types)	Illumina 450 K, EPIC	① Predict DNAmTL; ② Association of DNAmTL and the all‐cause mortality, heart disease, smoking history, social factors, immune cells, etc.
	DunedinPoAm (Belsky et al. 2020)	2020	810/cross‐validation and bootstrap	European	46 CpGs	Whole blood	3790/European/Whole blood	Illumina 450 K, EPIC	① Calculate the rate of aging; ② Correlation between age acceleration and physical functioning, cognitive function, as well as subjective signs of aging.
	DeepMAge (Galkin et al. 2021)	2021	4930/cross‐validation	Multiracial	1000 CpGs	Peripheral blood	1293/NA/Peripheral blood	Illumina 27 K, 450 K	① Predict chronological age; ② Association of chronological age with health‐related diseases.
	GrimAge2 (Lu et al. 2022)	2022	1833/711	European/Multiracial	1030 CpGs	Whole blood	13,831/Multiracial/Whole blood, saliva	Illumina 450 K, EPIC	① Predict mortality, morbidity and biological age, and calculate age acceleration; ② Correlation between biological age and all‐cause mortality, disease incidence risk.
	DunedinPACE (Belsky et al. 2022)	2022	817/cross‐validation and bootstrap	European	173 CpGs	Whole blood	6111/European/Whole blood	Illumina 450 K, EPIC	① Calculate the rate of aging; ② Correlation between age acceleration and physical functioning, cognitive function, as well as subjective signs of aging
	Precious1GPT (Urban et al. 2023)	2023	Methylation: 8374/4019; RNA‐seq: 12,453/2730	European and American	14,000 CpGs	Multiple tissues (26 types)	160/NA/Multiple tissues (6+ types)	Illumina 450 K, EPIC	① Predict chronological age; ② iPSCs can reverse age‐related methylation changes.
The Second‐generation Epigenetic Clock	DNAmFitAge Clock (McGreevy et al. 2023)	2023	2957/cross‐validation	Multiracial	627 CpGs	Whole blood	9069/Multiracial/ Whole blood, urine	Illumina 450 K, EPIC	① Predict biological age and calculate age acceleration; ② Correlation between biological age and physical parameters, mortality rate, risk of coronary heart disease.
	cAge; bAge (Bernabeu et al. 2023)	2023	cAge: 24,674/6261; bAge: 18,365/NA	Multiracial	cAge: 2330 CpGs; bAge: 35 CpGs	Whole blood, saliva/Whole blood	cAge: 2711/European/Whole blood, saliva; bAge: 4134/European/ Whole blood	Illumina 450 K, EPIC	① Predict biological age, chronological age and calculate bAge acceleration; ② Correlation between cAge and short‐term aging trajectory;③ Association of bAge with all‐cause mortality; ④ Identification of aging‐related biomarkers.
	Wrinkle Predictor; Visual Facial Skin Age Clock; VisAgeX (Bienkowska et al. 2023)	2023	302/76	European	Wrinkle Predictor/Visual Facial Skin Aging Clock: 794,441 CpGs	Epidermal tissue of the human forearm	Set1: 51/European/Epidermis. Set2: 25/European/Epidermis	Illumina EPIC, HiSeq	① Assess the rate of facial aging, categorize aging phenotypes, and uncover the underlying biological pathways.
	ResnetAge (Shi et al. 2023)	2023	11,933/3580	NA	22,278 CpGs	Multiple tissues (9 types)	1202/Multiracial/whole blood, saliva, buccal, T cell, uterine cervix, serum	llumina 27 K, 450 K	① Predict chronological age; ② Correlation between chronological age and tissue function as well as the risk of AD.
	iCAS‐DNAmAge (Zheng et al. 2024)	2024	250/125	Asian	65 CpGs	Whole blood	1070/Asian/ Whole blood	Illumina EPIC, EPIC v2.0	① Predict biological age, chronological age and calculate average age acceleration; ② Association of chronological age with hypertension and inflammation‐related diseases.
	CausAge; DamAge; AdaptAge (Ying et al. 2024)	2024	2664/10 FCV	European	CausAge: 586 CpGs; DamAge: 1090 CpGs; AdaptAge: 1000 CpGs	Whole blood	Set 1: 4651/European/ Whole blood; Set 2: 4651/European/ Whole blood, skin, oral mucosa	Illumina 450 K, EPIC	① Predict biological age and calculate age acceleration; ② Separate age‐related damages from adaptive changes; ③ Link parameters to mortality, verify short‐term intervention efficacy, and map aging‐related pathways.
	Retroelement‐Age (Ndhlovu et al. 2024)	2024	10,138/2532	NA	Retroelement‐Age V1: 1317 CpGs; V2: 1378 CpGs	Whole blood	14,152/NA/Multiple tissues (13 types of human tissues, 59 types of mammalian tissues)	Illumina 450 K, EPIC	① Predict chronological age; ② Assess the impact of HIV infection and intervention on aging; ③ Monitor Epigenetic Reversal in Transient Reprogramming.
	CheekAge (Shokhirev et al. 2024)	2024	8045/10 FCV	American	NA	Oral buccal mucosal cells	271/NA/Multiple tissues (6 types)	Illumina EPIC	① Predict chronological age and calculate age acceleration; ② Correlation between chronological age and lifestyle factors as well as disease status.
	IntrinClock (Tomusiak et al. 2024)	2024	9104/2994	NA	381 CpGs	PBMCs, brian, skin, saliva	623/NA/PBMCs, fibroblasts, immune cells	Illumina EPIC	①Predict chronological age and calculate average age acceleration; ② Correlation between chronological age and cellular functions; ③ Association of age acceleration with HIV and COVID.
	NCAE‐CombClock; NCAE‐Age (Martínez‐Enguita et al. 2024)	2025	NCAE‐CombClock: 14,181/3545; NCAE‐Age: 1404/330	European	NCAE‐CombClock: 1871 CpGs; NCAE‐Age: 1000 CpGs	Multiple tissues (6 types)	259/NA/Whole blood, saliva, oral epidermis, muscle, semen	Illumina 450 K, EPIC	① Predict chronological age; ② Identify aging‐related biological pathways; ③ Association of age acceleration with the risk of Crohn's disease.
	InflammAge (Schmunk et al. 2025)	2025	338/61	NA	3788 CpGs	Saliva	18,865/European/ Whole blood, saliva	Illumina EPIC	① Predict chronological age and calculate average age acceleration; ② Age acceleration linked to: mortality, cancer, circulatory & infectious diseases, immunosenescence, and lifestyle.
The Third‐generation Epigenetic Clock	rDNAm Age Clock (Wang and Lemos 2019)	2019	153 mouse, 80 canine/10 FCV; 8 humans/NA	mouse, canine/ mouse, canine, NA	mouse: 72 CpGs; cross species (mice and canine): 88 CpGs	Whole blood (mouse, canine)/Whole blood (mouse, canine), cell lines, skin (Human)	32 (mouse)/CR mouse/Whole blood, iPSC, liver	Illumina HiSeq2500	① Predict chronological age and calculate age acceleration; ② Correlation between age acceleration and nuclear function, and intervention response (CR, iPSC); ③ rDNA hypermethylation linked to breast cancer, MDS, and hippocampal anomalies in suicide decedents.
	Universal pan‐mammalian Epigenetic Clocks (Lu, Fei, et al. 2023)	2023	11,754/LOFO	185 species of mammals	Clock1: 736 CpGs; Clock2: 401 CpGs; Clock3: 387 CpGs	Multiple tissues (59 types)	Human cohort: 4661/Multiracial/ Whole blood, myelomoncyte; Mouse cohort: 428/NA/Multiple tissues (6 types)	Illumina Horvath Mammal MethylChip	① Predict chronological age and calculate age acceleration; ② Correlation between chronological age and DNAm function, chromatin function, developmental function; ③ Association of age acceleration with metabolic disease, nerve disease, cancer.
Trascriptomic Clock	Transcriptomic Aging Clock (Peters et al. 2015)	2015	7074/LOOCV	Multiracial	1497 Genes	Whole blood	7909/Multiracial/ Multiple tissues (6+ types)	Illumina HT12v3/4; Affymetrix Exon 1.0 ST	① Predict chronological age and calculate average age acceleration; ② Correlation between chronological age and gene function.
Trascriptomic Clock	Transcriptomic Aging Clock (Fleischer et al. 2018)	2018	133/LOOCV	NA	4852 Genes	Human dermal fibroblasts	22/NA/Human dermal fibroblasts	NA	① Predict chronological age and calculate average age acceleration; ② Association of biological age with the risk of HGPS.
	RE‐based Transcriptomic Aging Clock (LaRocca et al. 2020)	2020	133/NA	NA	1200 Genes	Human dermal fibroblasts	38/NA/Human dermal fibroblasts	Illumina HiSeq 2000/2500, NextSeq 500	① Predict chronological age; ② Correlation between chronological age and RE transcript levels; ③ Association of biological age with the risk of HGPS.
	Meta‐clock (Liu et al. 2020)	2020	2993/10 FCV	NA	DNAm: 1600 CpGs; Transcriptome: 8589 Genes	Whole blood	943+/NA/whole blood, DLPFC, 143B cell line, human dermal fibroblast cell line	Illumina 450 K	① Predict biological age; ② Association of biological age with health outcomes (cancer, AD, mortality).
	Transcriptomic Aging Clock (Holzscheck et al. 2021)	2021	620/267	European	4359 Genes	Epidermis	NA	Illumina HiSeq	① Predict chronological age and calculate age acceleration; ② Correlation between biological age and phenotypic aging as well as the risk of HGPS, skin diseases.
	Transcriptomic Aging Clock (Shokhirev and Johnson 2021)	2021	3060/10 FCV	NA	13,388 Genes	Multiple tissues (7 types)	NA	NA	① Predict chronological age; ② Correlation between biological age and biological function as well as the risk of CVD, cancers, etc.
	scRNA‐seq‐based Aging Clock (Zhu, Chen, et al. 2023)	2023	45/Loocv	Asian	3244 Genes	PBMCs	15+/Asian/PBMCs	Illumina HiSeq PE150	① Predict chronological age and calculate age acceleration; ② Correlation between biological age and aging function as well as the risk of COVID‐19 and SLE.
	MultiTIMER (Jung et al. 2023)	2023	3000+/FCV	NA	818 Genes	Multiple tissues (6+ types)	NA	NA	① Predict chronological age; ② Correlation between chronological age and cellular function as well as anti‐aging intervention.
	Human blood transcriptome‐based aging clock (Duran and Tsurumi 2025)	2025	105/24	Multiracial	12,417 Genes	PBMCs	42/Multiracial/PBMCs	HG‐U133A	① Predict chronological age and calculate age acceleration; ② Correlation between chronological age and gene expression function as well as the risk of age‐related diseases.
Proteomic Clock	Proteomic Aging Clock (Lehallier et al. 2020)	2020	2178/1123	European	476 proteins	Plasma	①171/European/Plasma; ②47/European/Plasma	NA	① Predict chronological age and calculate age acceleration; ② Correlation between chronological age and protein function as well as the risk of age‐related diseases.
	iAge (Sayed et al. 2021)	2021	1001/5 FCV	Multiracial	50 blood immune proteins; 397 genes; 935 cell phenotypes	Serum, PBMCs	①97/NA/Peripheral Blood; ②37/European/Plasma; ③2290/NA/Peripheral Blood	Illumina PE150	① Predict biological age and calculate age acceleration; ② Correlation between biological age and immune/cardiovascular function; ③ Association of biological age with age‐related diseases/frailty.
	ImmuneAge (Jia et al. 2023)	2023	43,096	Asian	36 Immune parameters	Peripheral blood	153/Asian/Peripheral blood	NA	① Predict chronological age; ② Correlation between age and immune cell function; ③ Association between biological age and disease risk, gender differences.
	PAC Clock (Kuo et al. 2024)	2024	37,115/15,906	Multiracial	128 Plasma proteins	Plasma	NA	NA	① Predict biological age and calculate average age acceleration; ② Biological age correlation with health and mortality.
	Organ‐specific Aging Models (Goeminne et al. 2025)	2025	35,962/8990	Multiracial	2916 Plasma proteins	Plasma, CSF	① 921/Multiracial/Plasma; ② 383/NA/Plasma; ③ 212/NA/Plasma and CSF; ④ 31/NA/Plasma	NA	① Predict chronological age, biological age and the rate of aging; ② Correlation between biological age and lifestyle physiological functions, organ‐specific diseases as well as all‐cause mortality.
	Immune Age Prediction Model (Heming et al. 2025)	2025	8790/10 FCV	European	23 Immune cell subsets; 37 CSF cell parameters, 7 CSF biochemical parameters	CSF, Peripheral blood	3201/European/CSF	NA	① Predict chronological age; ② Correlation between chronological age and immune function, disease groups as well as neurological disease classification.
Microbiome Clock	Microbiome aging clock (Galkin et al. 2020)	2020	Host level: 1165/10 FCV; Sample level: 3665/5 FCV	Multiracial	1673 species‐level microorganisms, 41 key species	Stool	Health dataset: 402/Multiracial/Stool; T1D dataset: 34/NA/ Stool	Illumina HiSeq 2000, GAII, GAIIx	① Predict chronological age and calculate average age acceleration; ② Correlation between chronological age and anti‐inflammatory function and metabolic function; ③ Association of gut microbiome aging with T1D and inflammation‐related diseases.
Microbiome Clock	Gut Microbiome Aging Clock (Chen et al. 2022)	2022	2604/5 FCV	Multiracial	904 gut microbiota species, 468 metabolic pathways	Stool	NA	Illumina HiSeq, MiSeq	① Predict chronological age; ② Correlation between chronological age and gut microbiome function; ③ Association of biological age with malnutrition, inflammation and neurodegenerative diseases.
	Gut Microbiome Aging Clock (Gopu et al. 2024)	2024	Microbiome: 78,637/FCV; Transcriptome: 1494/NCV	Multiracial	5010 gut microbiomes; 6576 KEGG ortholog features; 18,457 blood genes	Stool, Whole blood	1416/Multiracial/Stool	Illumina NextSeq 500, NovaSeq 6000	① Predict chronological age and calculate average age acceleration; ② Correlation between chronological age and microbiome/human gene function; ③ Association of biological age with lifestyle and disease.
	Multi‐biological data‐based Age Predictor (Han et al. 2025)	2025	183/10 FCV	Asian	60 Brain MRI features, 10 microbiota, 7 blood biochemical indicators	Brain MRI, Stool, Peripheral blood	NA	Illumina MiSeq	① Predict chronological age and calculate average age acceleration; ② Correlation between biological age and cognitive/mental function as well as the risk of schizophrenia.
Metabolome Clock	Pregnancy Metabolic Clock (Liang et al. 2020)	2020	507/10 FCV	European	9651 Plasma metabolites	Plasma	32/European/Plasma	NA	① Predict the actual gestational age and calculate the average gestational age acceleration; ② Correlation between chronological age and metabolic function, pregnancy complications.
	MetaboAge (van den Akker et al. 2020)	2020	18,716/5 FCV	European	56 Serum metabolite variables	Serum	**PROSPER queue**: 5326/European/serum; LLS_SIBS queue: 326/ European/serum	NA	① Predict chronological age and calculate average age acceleration; ② Correlation between chronological age and metabolic function, the risk of CVD, mortality rates as well as T2D.
	Cerebrospinal Fluid Metabolomic Aging Clock (Hwangbo et al. 2022)	2022	85/LOOCV	American	108 metabolomic features, 6735 global metabolomic features, 854 GOT‐MS features, 1070 lipid features	CSF	113/American/CSF	NA	① Predict chronological age and calculate age acceleration; ② Correlation between chronological age and metabolic function, the risk of AD as well as PD.
	HAM Index (Hamsanathan et al. 2024)	2024	196/10 FCV	NA	25 Serum metabolites	Serum	NA	NA	① Predict biological age; ② Correlation between chronological age and physical function kidney function, CVD as well as inflammation‐related diseases.
	MileAge (Mutz et al. 2024)	2024	101,359/10*5 NCV	European	168 Plasma metabolites	Plasma	NA	Nightingale Health	① Predict chronological age and calculate average age acceleration; ② Correlation between chronological age and physical function, the risk of chronic disease, all‐cause mortality.
	MetaboHealth (van Holstein et al. 2024)	2024	192	European	58 Serum metabolites	Serum	NA	NA	① Predict biological age and mortality.
Single‐cell Clock	SenCID (Tao et al. 2024)	2024	602/leave‐one‐cell‐type‐out	Asian	1290 SRGs	Multiple tissues (30 types)	NA	NA	① Predict biological age; ② Correlation between biological age and cell function, the risk of COVID‐19, IPF, as well as OA.
	Microglia scRNA‐seq Aging Clock (Stanley et al. 2025)	2025	89/54,857, leave‐one‐cell‐type‐out	NA	Hammond dataset: 51,000 gene features; Buckley dataset: 42,000 gene features; Kracht dataset: 21,905 gene features	Microglia in human/mouse brain tissue	NA	Illumina NextSeq 500, HiSeq 4000, NvaSeq 6000	① Predict chronological age; ② Correlation between chronological age and microglia function, the risk of neuropsychiatric disease.

Abbreviations: AA, age acceleration; AD, Alzheimer's disease; BA, biological age; BEC, buccal epithelial cells; BMI, body mass index; CA, chronological age; COVID‐19, Coronavirus disease 2019; CPC, control placental clock; CpGm, CpG methylation; CPGs, cytosine‐phosphate‐guanine; CR, calorie restriction; CSF, cerebrospinal fluid; CVD, cardiovascular disease; DLPFC, dorsolateral prefrontal cortex; DunedinPACE, dunedin pace of aging calculated from the epigenome; EEAA, extrinsic epigenetic age acceleration; EPI, epiglottis; FCV, fold cross validation; GOT‐MS, global optimized targeted mass spectrometry; HGPS, Hutchinson Gilford Progeria Syndrome; HIV, human immunodeficiency virus; IBS, irritable bowel syndrome; IEAA, intrinsic epigenetic age acceleration; IPF, idiopathic pulmonary fibrosis; iPSCs, induced pluripotent stem cells; IVD, intervertebral discs; LC–MS, liquid chromatography‐mass spectrometry; LOFO, leave one point for cross‐validation; LOOCV, leave‐one‐out cross‐validation; MC, molecular clock; Mlp‐DDR, multilayer perceptron‐ degenerative disease risk prediction; MSCAP, Multi‐Scale Convolutional Age prediction; NCAE, network‐coherent autoencoder; NCV, nested cross‐validation; OA, osteoarthritis; PBL, peripheral blood lymphocytes; PBMC, peripheral blood monocytes; PD, Parkinson's disease; PMA, post‐menstrual age; PNA, post‐natal age; RE, repetitive element; RPC, robust placental clock; SEA, sperm epigenetic age; SLE, systemic lupus erythematosus; SRGs, senescence‐related genes; T1D, type 1 diabetes; T2D, type 2 diabetes; UPM, Universal PaceMaker; Y‐CpGs, Y‐chromosomal CpGs.

**TABLE 2:** The summary of the evolution of methodologies for biological age in aging clock.

Model architecture	Clock name	Year	MAE in training dataset (years)	MAE in testing dataset (years)	PCC in training dataset	PCC in testing dataset
*Standard Linear Regression*						
Multiple Linear Regression	WeidnerAge (Weidner et al. 2014)	2014	MAE ≤ 7.2 years	MAE ≤ 5.6 years	NA	NA
	VidalBraloAge (Vidal‐Bralo et al. 2016)	2016	NA	NA	0.68	0.45
	MetaboAge (van den Akker et al. 2020)	2020	7.3 years	NA	0.654	NA
	BrainAge (Monti et al. 2020)	2020	CamCAN: ≈8 years; HCP: ≈12–14 years; ATR: 11.5–14.5 years		NA	NA
	PhysiAge (Janssens et al. 2023)	2023	MAE ≤ 11.72 years	MAE ≤ 11.79 years	0.906	0.916
Least Squares Regression	epiTOC (Yang et al. 2016)	2016	NA	NA	B‐cell samples: 0.61; CD4+ T‐cell sample: 0.52; Monocyte samples: 0.74	
Klemera‐Doubal	DNAmFitAge Clock (McGreevy et al. 2023)	2023	NA	NA	NA	0.77
*Regularized Linear Regression*						
Ridge Regression	Robust Epigenetic Model (Montesanto et al. 2020)	2020	5.13 years	4.5 years	0.74	0.9
	Proteomic Aging Clock (Lehallier et al. 2020)	2020	1.84 years	2.44 years	0.98	0.96
Elastic Net Regression	Horvath Clock (Horvath 2013)	2013	2.9 years	3.6 years	0.97	0.96
	Hannum Clock (Hannum et al. 2013)	2013	3.9 years	4.9 years	0.96	0.91
	DNAm GA (Knight et al. 2016)	2016	1.24 weeks	1.49 weeks	0.99	0.91
	ZhangAge (Zhang et al. 2019)	2019	NA	NA	0.99	0.91
	Placental Clock (Lee et al. 2019)	2019	RPC: 0.96 weeks; CPC: 1.02 weeks; Refined RPC: 1.49 weeks	NA	NA	RPC: 0.99; CPC: 0.98; Refined RPC: 0.98
	NEOAge (Graw et al. 2021)	2021	450 k‐PMA NEOage: MAE < 1.28 weeks; 450 k‐PNA NEOage: MAE < 1.63 weeks	450 k‐PMA NEOage: MAE < 1.09 weeks; 450 k‐PNA NEOage: MAE < 1.55 weeks	450 k‐PMA NEOage: 0.93; 450 k‐PNA NEOage: 0.93	450 k‐PMA NEOage: 0.61; 450 k‐PNA NEOage: 0.76
	Pan‐tissue Methylation Aging Clock (Vijayakumar and Cho 2022)	2022	1.62 years	2.8 years	0.99	0.97
	Centenarian Clock (Dec et al. 2023)	2023	ENCen40+: 2.34–3.62 years; NNCen40+: 2.28–3.00 years; ENCen100+: 1.79–34.65 years	ENCen40+: 10.64 years; NNCen40+: 26.85 years; ENCen100+: 58.95 years	ENCen40+: 0.592–0.961; NNCen40+: 0.636–0.959; ENCen100+: 0.381–0.604	ENCen40+: 0.945; NNCen40+: 0.740; ENCen100+: 0.503
	Visual Facial Skin Age Clock; VisAgeX (Bienkowska et al. 2023)	2023	Visual Facial Skin Age Clock: 6.54 years; VisAgeX: 6.17 years	Visual Facial Skin Age Clock: 5.76 years; VisAgeX: 4.67 years	Visual Facial Skin Age Clock: 0.84; VisAgeX: 0.30	Visual Facial Skin Age Clock: 0.91; VisAgeX: 0.48
	TWBAge, TWBhAge (Huang et al. 2025)	2025	TWBAge: MAE < 2.50 years TWBhAge: MAE < 2.51 years		TWBAge: 0.95 TWBhAge: 0.95	
	PhenoAge (Levine et al. 2018)	2018	NA	NA	0.71	0.62–0.78
	GrimAge (Lu, Quach, et al. 2019)	2019	NA	NA	0.82	≥ 0.79
Elastic Net Regression	DunedinPoAm (Belsky et al. 2020)	2020	NA	NA	0.56	0.33
	ipAGE (Yusipov et al. 2022)	2022	6.82 years	7.27 years	0.79	NA
	cAge (Bernabeu et al. 2023)	2023	cAge: 2.3 years	cAge: 1.74 years	cAge: 0.96	cAge: 0.96
	iCAS‐DNAmAge (Zheng et al. 2024)	2024	3.45 years	4.37 years	0.97	0.77
	CausAge; DamAge; AdaptAge (Ying et al. 2024)	2024	CausAge: 3.08 years; DamAge: 3.84 years; AdaptAge: 4.93 years		CausAge: 0.82; DamAge: 0.86; AdaptAge: 0.59	
	Retroelement‐Age (Ndhlovu et al. 2024)	2024	Retroelement‐Age V1: 2.57 years; Retroelement‐Age V2: 1.87 years	Retroelement‐Age V1: 3.81 years; Retroelement‐Age V2: 2.08 years	Retroelement‐Age V1: 0.95 Retroelement‐Age V2: 0.97	Retroelement‐Age V1: 0.89; Retroelement‐Age V2: 0.96
	IntrinClock (Tomusiak et al. 2024)	2024	3.83 years	NA	0.972	NA
	InflammAge (Schmunk et al. 2025)	2025	3.83 years	4.2 years	0.93	0.94
*Dimensionality Reduction Regression*						
Partial Least Squares Regression	Human PBMC scRNA‐seq‐based Aging Clock (Zhu, Chen, et al. 2023)	2023	MAE > 8.36 years	10.06 (Chinese Young Cohort), 10.91 (Wuhan Cohort), 10.16 (Japanese Old Cohort)	0.88	0.43 (Chinese Young Cohort), 0.69 (Wuhan Cohort), 0.28 (Japanese Old Cohort)
	PLSR BrainAge (Guan et al. 2024)	2024	7.90–11.33 years	NA	0.72–0.87	NA
*Generalized Linear Models*						
GLM + Elastic Net regularization	MultiTIMER (Jung et al. 2023)	2023	8.2	NA	0.87	NA
Deep GLM	NC‐BA (Deng et al. 2025)	2025	5.33 years	7.12 years	0.58	0.57
*Nonlinear Regression*						
Gaussian Process Regression	IVD/EPI Molecular Clock‐Based Age Prediction Model (Becker et al. 2020)	2020	IVD: 6.3 years; EPI: 5.5 years; IVD + EPI: 4.0 years	NA	IVD: 0.86; EPI: 0.91; IVD + EPI: 0.95	NA
	GP‐age (Varshavsky et al. 2023)	2023	2.08 years	Internal: 2.10 years; External: 2.24 years	0.97	0.97
*Rule‐Based Regression*						
Cubist Rule‐Based Regression	MileAge (Mutz et al. 2024)	2024	5.42 years	5.42 years	0.81	0.57
*Support Vector Regression*						
	Paparazzo Clock (Paparazzo et al. 2022)	2022	4.95 years (only ELOVL2); 4.45 years (ELOVL2 + sjTREC)	4.95 years (only ELOVL2); 4.43 years (ELOVL2 + sjTREC)	0.90 (only ELOVL2); 0.93 years (ELOVL2 + sjTREC)	NA
	brainAGE (Yu, Cui, et al. 2024)	2024	Age group of 5–40 years: 3.53 years (female); 3.60 years (Male); 40–90 years: 4.45 years (Female), 4.09 years (Male)	Total 5.28 years	Age group of 5–40 years: 0.83 (female), 0.84 (male); 40–90 years: 0.86 (female), 0.87 (male)	*r* = 0.68
*Stacking*						
Multiple base learner+Linear Regression	Gut Microbiome Aging Clock (Chen et al. 2022)	2022	8.33 years	NA	NA	NA
Simulated Annealing + Ensemble ML	CheekAge (Shokhirev et al. 2024)	2024	3.22 years	3.48 years	0.93	0.92
*Boosting*						
XGBoost regression	BrainAge (Beck et al. 2022)	2022	NA	T1‐based brain age model: 0.72; DTI‐based brain age model: 0.73	NA	NA
	XGBAge (Shim et al. 2024)	2024	NA	4.98 years	NA	0.6
*AutoML*						
SuperLearner	Childhood SL PCA Clock (Khodasevich et al. 2025)	2025	0.66 years	0.50 years	0.96	0.95
*Convolutional Neural Network*						
CNN	BrainAge (Cole et al. 2017)	2017	NA	CNN‐GM: 4.16 years; CNN‐Raw: 4.65 years; GPR‐GM: 4.66 years		CNN‐GM: *r* = 0.96; CNN‐Raw: *r* = 0.94; GPR‐GM: *r* = 0.95
	FacialAge (Wan et al. 2018)	2018	3.01 years (MORPH‐II)	3.30 years (CACD)	NA	NA
	FaceCnnAge (Xia et al. 2020)	2020	2.79 years	3.15 years	0.96	NA
	CXR‐Age (Raghu et al. 2021)	2021	6.24 years	NA	NA	Female: 0.80; Male: 0.84
CNN + DNN	PhotoAgeClock (Bobrov et al. 2018)	2018	1.92 years	2.3 years	NA	0.96
*Recurrent Neural Network*						
ConvLSTM	ConvLSTM (Rahman and Adjeroh 2019)	2019	λ = 0.9: 5.63 years	λ = 0.9: 13.4 years	λ = 0.9: 0.94	λ = 0.9: 0.55
*Deep Neural Network*						
	MethylNet (Levy et al. 2020)	2020	NA	3 years	0.96	0.96
	DeepMAge (Galkin et al. 2021)	2021	3.8 years	3.8 years	0.98	0.97
	iAge (Sayed et al. 2021)	2021	15.2 years	NA	0.78	NA
	Age‐Net (Armanious et al. 2021)	2021	NA	2.0–3.6 years	NA	0.86–0.98 years
	EchoAGE (Kobelyatskaya, Guvatova, et al. 2024)	2024	3.26 years	3.05 years	0.95	0.95
	AcidAGE (Kobelyatskaya, Isaev, et al. 2024)	2024	Comprehensive Model: 4.26 years; Simplified Model: 5.46 years	Comprehensive Model: 5.10 years; Simplified Model: 6.01 years	Comprehensive Model: 0.84	NA
	PerSEClock (Zhao et al. 2024)	2024	2.08 years	3.10 years	0.95	0.93
	NCAE‐CombClock; NCAE‐Age (Martínez‐Enguita et al. 2024)	2024	NCAE‐CombClock: 1.96 years; NCAE‐Age: 3.13 years	NCAE‐CombClock: 1.74 years; NCAE‐Age: 2.36 years	NCAE‐CombClock: 0.99; NCAE‐Age: 0.98	NCAE‐CombClock: 0.98; NCAE‐Age: 0.91
	ECG‐BA (Liu, Kuo, et al. 2025)	2025	6.25 years	6.16 years	0.7	0.7
*Artificial Neural Network*						
	Transcriptomic Aging Clock (Holzscheck et al. 2021)	2021	4.7 years	4.4 years	NA	NA
*Convolutional Neural Network*						
	Retinal age (Zhu, Shi, et al. 2023)	2023	3.55 years	3.01 years	0.81	NA
	EyeAge (Ahadi et al. 2023)	2023	Actual age 2.86 years, perceived age 2.9 years	3.30 years (CACD)	0.962 actual age; 0.939 perceived age	0.87
	BrainAge (Yin et al. 2023)	2023	NA	Health group: 2.41 years for men and 2.23 years for women	NA	Health group:*r* = 0.98
	BrainAge (Hu et al. 2023)	2023	ResNet‐18 (raw data): 67.66 days; SVR (raw data vectors): 69.46 days	NA	ResNet‐18 (raw data): *r* = 0.9118; SVR (raw data vector): Pearson's *r* = 0.9177	NA
	Age Estimate Model (Kerber et al. 2023)	2023	5.76 ± 5.17 years	6.50 ± 5.18 years	*R* ^2^ = 0.84	*R* ^2^ = 0.74
	ResnetAge (Shi et al. 2023)	2023	1.29 years	3.24 years	0.99	0.98
	ECG‐Age, ECG‐Mort1Y and ECG‐Mort5Y (Cho et al. 2024)	2024	Internal validation: 7.3 years, external validation: 10.7 years	NA	Internal validation: *R* = 0.89, External validation: *R* = 0.85	*r* = 0.852 (*p* < 0.001)
	ThermoFace (Yu, Zhou, et al. 2024)	2024	5.08 years (male), 5.17 years (female)	4.40 years (male), 5.18 years (female)	0.9	0.91 for the PLSR model and 0.86 for the CNN model
	MSCAP; Mlp‐DDR (Zhang, Cai, et al. 2024)	2024	NA	NA	NA	NA
	AI‐ECG (Evans et al. 2025)	2025	NA	Internal validation: 9.13 years; external validation: 7.65 years; age‐matched group (44–85 years): 8.24 years	NA	Internal validation: *r* = 0.721; external validation: *r* = 0.457; age‐matched group: *r* = 0.557
	Age Prediction Model Using Fundus Images (Tanito and Koyama 2025)	2025	NA	Root Mean Square Error: 5.06 years	NA	NA
*Transformer*						
	Precious1GPT (Urban et al. 2023)	2023	DNA methylation: 4.227 years; transcriptome: 6.287 years; merger: 5.622 years	DNA methylation: 4.227 years; transcriptome: 6.287 years; merger: 5.622 years	DNA methylation: *R* ^2^ = 0.934; transcriptome: *R* ^2^ = 0.584; combined: *R* ^2^ = 0.807	NA

Abbreviations: ATR, Advanced Telecommunications Research Institute International; CACD, cross‐age celebrity dataset; CamCAN, Cambridge Center for Aging and Neuroscience; CAS, Chinese Academy of Sciences; CNN, Convolutional Neural Network; CPC, Control Placental Clock; DTI, Diffusion Tensor Imaging; ELOVL2, ELOVL Fatty Acid Elongase 2; ENCen100+, Elastic Net Centenarian Clock for individuals aged 100 and above; ENCen40+, Elastic Net Centenarian Clock for individuals aged 40 and above; EPI, epiglottis; GM, gray matter; GPR, Gaussian Processes Regression; HCP, Human Connectome Project; IVD, Intervertebral Discs; NCAE, Network‐Coherent Autoencoder; NNCen40+, Neural Network Centenarian Clock for individuals aged 40 and above; PCA, Principal Component Analysis; PLSR, Partial Least Squares Regression; PMA, post‐menstrual age; PNA, post‐natal age; RPC, Robust Placental Clock; sj‐TREC, Signal Joint T‐Cell Receptor Rearrangement Excision Circles; SL, super learner; TWB, Taiwan Biobank.

By revealing conserved epigenetic signatures of aging across mammals with markedly different lifespans, the third‐generation cross‐mammalian epigenetic clock supports a model in which aging is driven, at least in part, by a conserved late‐developmental program rather than exclusively by stochastic cellular damage (Gems et al. 2024). Consistent with this framework, third‐generation epigenetic clocks can estimate chronological age across multiple species, thereby creating a bridge between animal model research and human applications and accelerating clinical translation (Tangili et al. 2023; Kusters and Horvath 2025). A representative example is the universal mammalian epigenetic clock developed by Lu, Brbić, et al. (2023) which was designed to apply across mammalian species and tissue types and was trained on 11,754 samples from 185 species and 59 tissue types.

### 2.2 Transcriptomic Clock

Using high‐throughput transcriptome sequencing, researchers have developed transcriptomic aging clocks by systematically examining genome‐wide changes in gene expression (Horvath et al. 2022; Sun et al. 2025). Unlike more stable epigenetic markers, transcriptomic clocks directly indicate a cell's current functional state and pathway activity, making them highly dynamic (Meyer and Schumacher 2021; Zakar‐Polyák et al. 2024). Although they operate on different molecular layers, both transcriptomic and epigenetic clocks aim to go beyond chronological age to reveal the actual biological aging process. There are mechanistic connections between the two, as signals captured by some epigenetic clocks can be understood through transcriptional changes in metabolic and immune pathways (Peters et al. 2015; Liu et al. 2020).

Early studies primarily relied on large‐cohort analyses to identify significant links between gene expression and aging, then used these features for age prediction (Mathys et al. 2019; Solé‐Boldo et al. 2020). Peters et al. (2015) developed the first human blood transcriptomic aging clock using large‐scale microarray data. They identified 1497 age‐related differentially expressed genes and demonstrated their enrichment at CpG methylation sites in enhancer and insulator regions, indicating a coordinated aging process between epigenetic and transcriptional regulation. Further studies incorporated multi‐tissue and multi‐state samples, revealing notable tissue and physiological differences in transcriptomic aging signatures. Fleischer et al. (2018) examined human dermal fibroblasts and developed an integrated classifier that predicts chronological age in healthy individuals. Importantly, they demonstrated for the first time at the transcriptomic level that patients with progeria show signs of accelerated aging, providing a valuable tool for researching pathological aging. Similarly, Shokhirev and Johnson (2021) combined 3060 multi‐tissue RNA‐seq samples to create tissue‐specific aging clocks, uncovering unique molecular aging mechanisms across different organs.

With advances in methodology, the focus of transcriptomic clocks has shifted from simple age prediction to functional interpretability. That is, linking gene expression changes to specific cellular processes. A notable research study is the MultiTIMER developed by Jung et al. (2023) which directly connects age prediction to 25 core cellular processes. This approach not only distinguishes age differences but also identifies which functional modules are dysregulated during aging.

Functionally oriented transcriptomic clocks have also advanced the study of non‐classical biomarkers of aging. LaRocca et al. (2020) identified transcriptional buildup of noncoding repetitive elements (REs) as a conserved hallmark of aging, with abnormal increases seen in progeria patients. Their RE‐based age prediction model outperformed the standard protein‐coding gene model, opening a new noncoding genomic dimension for transcriptomic aging clock development.

In recent years, the development of single‐cell technologies has transformed transcriptomic aging clock research. It outperforms traditional bulk sequencing by overcoming its averaging limitations, reveals cell‐type‐specific aging patterns for refined biological age assessment, and enables aging clocks to monitor overall aging trends while accurately capturing heterogeneous cellular changes during aging. Tao et al. (2024) developed a machine learning tool, SenCID, to identify six cell–type–specific senescence signatures at the single‐cell level. This method enables real‐time tracking of cellular aging processes during normal aging, chronic disease, and COVID‐19 progression, while also uncovering hierarchical regulatory factors of senescence. Once again, compared to static epigenetic markers, transcriptomic clocks can provide real‐time insights into cellular function and pathway activity. Similarly, Zhu, Chen, et al. (2023) performed a detailed analysis of single‐cell transcriptomes from PBMCs in 28 human subjects. They uncovered the molecular basis behind the youthful transcriptomic profiles of long‐lived individuals compared to their chronological age. Expanding into the central nervous system, Stanley et al. (2025) mapped microglial aging trajectories using single‐cell sequencing. They demonstrated that a transcriptomic aging clock could be accurately created using only low‐dimensional features of cell subpopulation frequencies. Significantly, this clock performed consistently across different datasets and sequencing platforms, emphasizing the reproducibility and potential for translation of single‐cell–based transcriptomic clocks.

### 2.3 Proteomic Clock

Proteomic aging clocks assess aging through protein expression patterns or post‐translational modifications (Kliuchnikova et al. 2024). Compared to epigenetic and transcriptomic clocks, proteins as the direct agents of physiological functions better reflect underlying aging processes (Lehallier et al. 2020), providing clinically relevant biomarkers for disease prognosis and tracking intervention outcomes. However, protein expression is more susceptible to influences from diet, environment, and other external factors, reducing its stability (Kuo et al. 2024). Lehallier et al. (2020) developed a highly accurate proteomic clock using 491 plasma proteins associated with age. This model accurately predicted chronological age and emphasized the importance of signal transduction and immune system proteins for aging prediction. Regular aerobic exercise lowered predicted biological age, suggesting that proteomic clocks can reflect lifestyle influences on aging.

Building on this, process‐specific proteomic clocks have further improved mechanistic insights. Sayed et al. (2021) developed the inflammatory aging clock (iAge), focusing on proteins associated with chronic inflammation. They identified CXCL9 as a key factor inducing endothelial senescence and impairing vascular function, directly linking cardiovascular aging to protein‐level mechanisms and highlighting potential therapeutic targets.

The latest advances have achieved organ‐level resolution. In a landmark study published in Cell Metabolism, Goeminne et al. (2025) used plasma proteomic data to develop multi‐organ aging clocks including the brain, arteries, liver, gut, kidneys, lungs, skin, and immune system. This research quantitatively connected chronic disease processes to organ‐specific accelerated aging. Ding et al. (2025) in Cell mapped a chronological atlas of organ aging, revealing that the aorta experiences the earliest and most significant proteomic network changes. They showed that senescence‐associated proteins (senoproteins) such as GAS6, SAP, and CXCL12 are secreted by vascular tissue and spread through the bloodstream, transmitting senescence‐associated secretory phenotype (SASP) signals systemically. This provided the first direct evidence that the vascular system acts as a central hub driving multi‐organ aging and that a key feature of aging is the systemic failure of proteostasis networks.

Collectively, proteomic clocks map the progressive spread of aging from local tissues to the bloodstream and eventually to the entire body. The breakdown of proteostasis in specific tissues produces senoproteins, which are released into the blood and act both as triggers and signals of aging, driving aging across different organs.

### 2.4 Microbiome and Metabolism Clock

The gut microbiome, often called the host's “second genome,” undergoes structural changes with age, highlighting the importance of host–microbe interactions in aging (Wilmanski et al. 2021). Microbiome‐based aging clocks provide the benefit of noninvasive sampling while reflecting the aging status of the gut microecosystem, offering unique mechanistic insights (Narasimhan et al. 2021). Galkin et al. (2020) analyzed metagenomic data from 4000 individuals across 10 public datasets and built the first quantitative microbiome aging clock, identifying genera like Bifidobacterium and *Akkermansia muciniphila* as strongly associated with age. Chen et al. (2022) included geographical factors and used multi‐view learning to combine taxonomic composition with metabolic pathway data, enhancing prediction accuracy and uncovered age‐related patterns such as shifts in *Finegoldia magna* abundance and changes in amino acid utilization. Building on this, Gopu et al. (2024) combined large‐scale metatranscriptomic and blood transcriptomic datasets, discovering links between lifestyle and biological age. They found that vegetarians showed younger microbial ages, and those with irritable bowel syndrome displayed accelerated aging. These results demonstrate the value of microbiome clocks for studying population differences and pathology.

Parallel progress has been made in metabolomic aging clocks. As direct indicators of physiological state, metabolomes hold a unique position among aging biomarkers (Huang, Chen, et al. 2025). Unlike other omics layers, metabolomics is closer to phenotypic outcomes, integrating signals from genes, environment, and lifestyle (Bauermeister et al. 2022), providing real‐time, functionally relevant insights into biological age (Deelen et al. 2019). van den Akker et al. (2020) developed MetaboAge, which predicted future cardiovascular disease risk and mortality in healthy populations while reflecting functional status in older adults, offering the first evidence that metabolomics can serve as a universal marker of overall aging. In pathological contexts, van Holstein et al. (2024) showed that the MetaboHealth was significantly linked to 1‐year mortality in older cancer patients. Metabolomic clocks have demonstrated flexibility in specific physiological states. Liang et al. (2020) created a pregnancy‐specific clock accurately predicting gestational age using just five plasma metabolites. Notably, deviations from ultrasound‐based estimates signal fetal growth status and delivery timing. The field has advanced from merely noting associations to understanding mechanisms. Hamsanathan et al. (2024) introduced the Healthy Aging Metabolomic (HAM) index, differentiating healthy from rapid agers and identifying lipid metabolism dysregulation as a significant factor in aging. Mutz et al. (2024) developed MileAge, confirming that differences between metabolomic age and chronological age are linked to established aging markers like telomere length and frailty.

At the research frontier, studies are progressing toward multimodal integration. Han et al. (2025) combined brain MRI, gut microbiome, and blood metabolomic data, showing in schizophrenia patients that differences in biological age negatively correlate with cognitive performance. This demonstrates how aging assessment is entering a new stage of multi‐omics integration, further expanding the applications of microbiome and metabolomic clocks in mechanistic aging research and clinical prognosis.

### 2.5 Function‐Oriented Aging Clock

Function‐oriented clocks measure physiological decline of organs or systems using imaging methods like MRI, CT, X‐ray, and photographs. Brain MRI can predict cognitive performance and dementia risk (Nowogrodzki 2024), chest CT relates to cardiovascular prognosis (Xu et al. 2024), and retinal fundus images are linked to mortality risk (Haugg et al. 2025). Organ‐specific AI models identify unique aging features for age prediction, while combining multiple modalities improves overall understanding for disease risk assessment and health management (Haugg et al. 2025).

Brain aging exhibits notable spatial variability, with faster aging in certain areas serving as early indicators of neurodegenerative diseases. Reduction in hippocampal volume is closely linked to age‐related cognitive decline, while lower gray matter density in the prefrontal cortex is associated with impaired working memory (Heo et al. 2025). Functional connectivity within the default mode network (DMN) also declines significantly with age during resting‐state conditions (Arnatkeviciute et al. 2023). Brain aging clocks typically rely on PET‐CT (Sai et al. 2025), structural MRI, and functional MRI (Erken and Shuang 2025; Whitman et al. 2025) to estimate brain age and aging patterns in patients with Parkinson's disease (PD) (Erken and Shuang 2025), Alzheimer's disease (AD) (Asif et al. 2025), and mild cognitive impairment (MCI) (Sai et al. 2025). Blood‐based biomarkers further enhance neuroimaging evaluation (Liu, You, et al. 2025). Chen et al. (2024) showed that PD patients experience accelerated aging in several regions, including the frontal and temporal cortices, limbic system, basal ganglia, thalamus, and cerebellum, revealing potential early intervention targets. Among them, neuroprotective strategies targeting the cerebellum and thalamic cortex might delay cognitive decline (Beheshti et al. 2024).

MCI indicates a prodromal stage of AD. As MCI progresses to AD, the gap between brain age and chronological age widens stepwise, reflecting increasing atrophy patterns (Ghaderi et al. 2025). In MCI, atrophy mainly affects the hippocampal CA4 subregion and dentate gyrus. While in AD, atrophy extends to neocortical areas such as the precuneus and inferior frontal gyrus, with faster degeneration of the hippocampal CA1 subregion (Darekar et al. 2025; Ghaderi et al. 2025). Notably, AD shows accelerated tissue loss: hippocampal atrophy occurs at 2.5 times the rate seen in healthy aging, with annual gray matter loss reaching 1.5%–2.0% compared to 0.5% in healthy individuals (Liu, Xu, et al. 2025). Sex‐specific trajectories further refine this picture. AD female patients show tau‐driven accelerated atrophy in the medial temporal lobe (3.5% annual loss, compared with 2.8% in males), along with increased posterior cingulate tau deposition and a brain age gap of +5.98 years. Male patients exhibit vascular‐driven pathology with uniform gray matter loss (1.8% annual loss vs. 1.3% in females), accelerated ventricular enlargement, and increased white matter hyperintensity volume, resulting in a brain age gap of +6.48 years (Yin et al. 2023; Yi et al. 2025). Importantly, brain age clocks have also been used with children. Hu et al. (2023) developed a pediatric brain developmental model (3D ResNet‐18), achieving high accuracy in predicting brain age in 0–3‐year‐old groups and measuring delayed brain maturation in preterm infants.

Skin aging involves collagen loss, elastin fiber fragmentation, and pigment deposition (Li 2023; Bar 2025), driven by both intrinsic aging and photoaging (Shin et al. 2019; Luo et al. 2026). Measurable changes in facial geometry can indicate aging progression. Mandibular contour relaxation and chin protrusion are key predictors of age in people over 40 (Wan et al. 2018). Deepening of the nasolabial fold is linked to maxillary retrusion, fat displacement, and dermal degeneration (Xia et al. 2020). The periorbital region serves as a sensitive marker of aging. Morphological changes in eye wrinkles strongly correlate with chronological age. Bobrov et al. (2018) developed the PhotoAgeClock, predicting age from periocular micro‐wrinkles with a mean error of 2.3 years across individuals aged 20–80. Similarly, retinal age models have shown that each additional year of retinal age difference corresponds to a 2% increase in all‐cause mortality risk (Zhu, Shi, et al. 2023). Furthermore, a reduced fractal dimension of retinal vasculature has been linked to brain aging–related genes such as ALKAL2, indicating a potential mechanism for cross‐organ aging (Ahadi et al. 2023). At the pathological level, patients with exfoliative glaucoma demonstrate accelerated retinal aging of approximately 2.13 years, positively correlating with advanced glycation end product (AGE) accumulation (Tanito and Koyama 2025).

Cardiovascular aging clocks reflect the dynamic interaction between electrophysiological remodeling and structural changes (Rabkin et al. 2016; Ribeiro et al. 2023). Age‐prediction models such as AI‐ECG and EchoAGE, based on electrocardiograms and echocardiography, effectively identify accelerated cardiac biological aging and are strongly linked to cardiovascular risk and clinical events (Shelly et al. 2023; Cho et al. 2024; Kobelyatskaya, Guvatova, et al. 2024). Additionally, functional biological age (fBioAge) models including measures like grip strength and lung function are notably associated with cognitive decline and other health outcomes, broadening the functional aspect of aging clocks beyond imaging and molecular signatures (Sternäng et al. 2019).

### 2.6 Multi‐Omics Aging Clocks

Different omics clocks capture distinct aging dimensions: epigenetic clocks reflect cumulative regulatory damage; transcriptomic clocks capture dynamic cellular states; proteomic/metabolomic clocks sense recent physiological disturbances; microbiome clocks integrate environmental and lifestyle effects. Although single‐omics clocks advance age prediction and disease risk assessment, they only capture partial aging signals. Studies show weak correlations across DNA methylation, transcriptomic, proteomic, and metabolomic clocks (Jansen et al. 2021), supporting the multidimensional heterogeneity of aging. Thus, multi‐omics clocks aim not merely to improve prediction accuracy, but to distinguish shared from omics‐specific aging signatures, thereby enhancing interpretation of functional decline, disease risk, and organ‐specific aging, and moving closer to underlying aging mechanisms (Jung et al. 2023).

Currently, multi‐omics integration strategies mainly encompass four approaches. First, parallel comparison constructs aging clocks for different omics separately within the same cohort and systematically compares their predictive performance and biological relevance. For example, the ORCADES cohort study simultaneously built DNA methylation, proteomic, and metabolomic aging clocks, revealing differences among omics layers in age prediction and disease associations (Macdonald‐Dunlop et al. 2022). Second, cross‐layer correlation analysis employs correlation and network‐based methods to identify shared aging signals across molecular layers. For example, genome‐wide association studies of epigenetic age acceleration and emerging analyses of proteomic aging traits suggest that part of clock variation may be rooted in heritable biological regulation (Lu et al. 2018). A recent study integrating genomic, transcriptomic, proteomic, and metabolomic data uncovered coordinated regulatory mechanisms for specific genes from transcription to protein levels in cardiovascular aging (Xiong et al. 2025). Third, joint modeling integrates multi‐omics data into a single machine learning framework to construct a unified aging clock, which can leverage complementary information across omics layers and identify key molecular biomarkers. The OMICmAge study, for instance, integrated DNA methylation, proteomic, metabolomic, and clinical data, achieving superior predictive performance compared with single‐omics models while maintaining good clinical accessibility (Chen et al. 2026). Fourth, multi‐scale integration further combines molecular omics with clinical phenotypes, medical imaging, and physiological function data to construct multilevel aging clocks from molecules to systems. The MULTIConsortium developed imaging‐based aging clocks using multi‐organ MRI data and linked them with plasma molecular data, identifying organ‐specific aging features and potential intervention targets (Cao et al. 2026).

From a performance perspective, multi‐omics aging clocks derive their value from integrating complementary biological signals rather than simply increasing model complexity. Studies such as OMICmAge and gtAge suggest that combining multiple omics layers can improve age prediction and better capture biological heterogeneity than single‐omics models (Xia et al. 2026; Chen et al. 2026). Multi‐scale frameworks that link molecular data with organ‐level imaging further extend this advantage by improving the detection of organ‐specific aging patterns (Cao et al. 2026). However, these gains are not universal. Current evidence remains limited and context‐dependent, and multi‐omics models do not necessarily outperform strong single‐omics clocks optimized for hard outcomes such as mortality or longevity. Their main advantage may therefore lie not only in predictive performance, but also in broader biological coverage, finer risk stratification, and stronger mechanistic interpretability.

## 3 Evolution of Modeling Methodologies

### 3.1 Early Statistical Models: Linear Regression and Basic Metric Applications (2013–2018)

**FIGURE 2:**
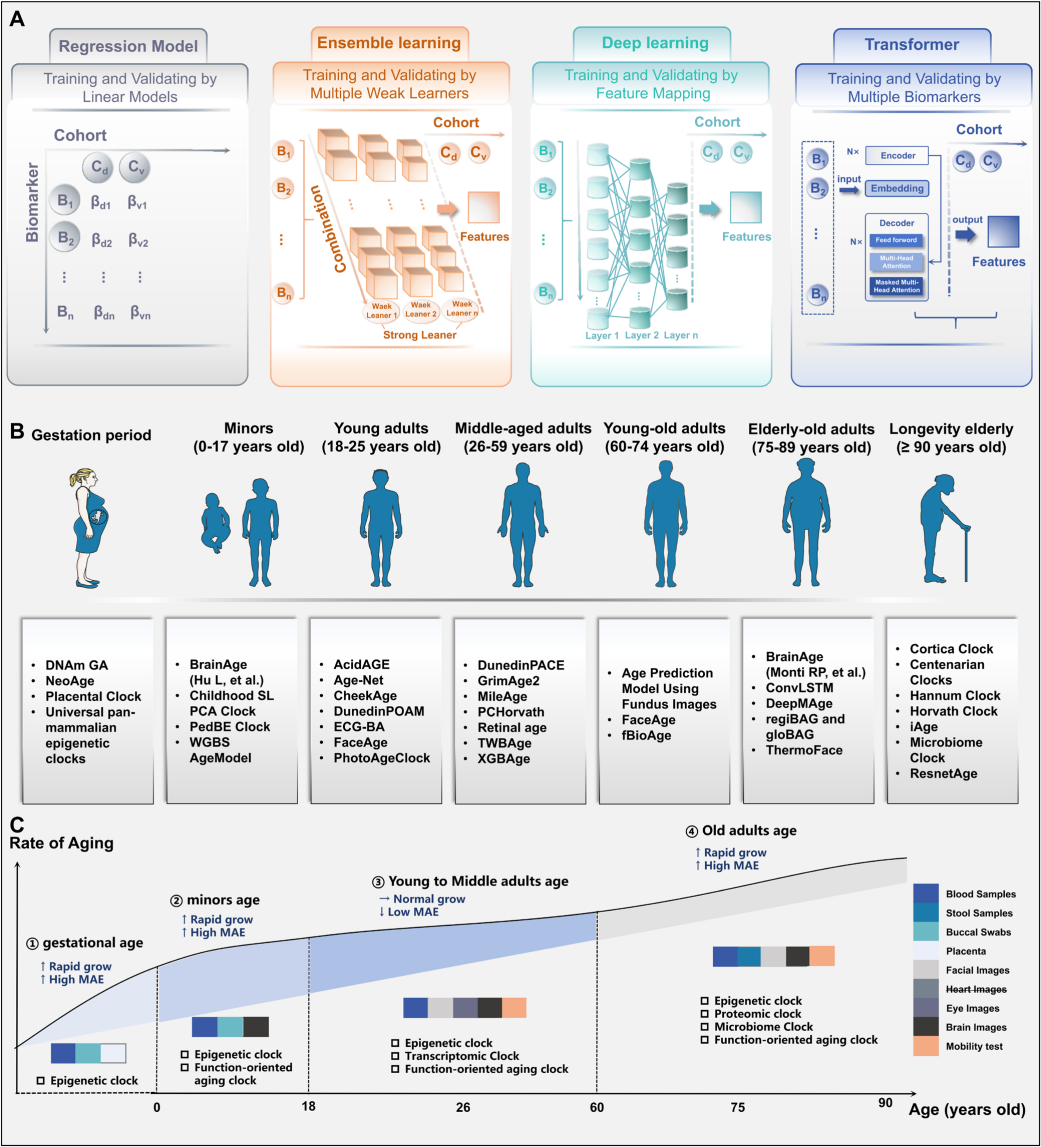
AI application in aging clocks. (A) Architecture comparison of aging clock models. (B) Aging clocks at different stages of human life. (C) Aging rate across life stages, associated aging clock categories, and sample modalities. Panel (A) shows Cd = Cohort for developing model; Cv = Cohort for validating model; B1…Bn = Biomarker 1 through *n*; Layer1…Layern = Layer 1 through *n*. MAE, mean absolute error.

Early research on aging clocks primarily relied on traditional statistical models such as linear regression. Among these, Elastic Net regression, an extension of linear regression, became the most widely used analytical tool in the field. This model incorporates both L1 and L2 regularization, automatically performs feature selection, prevents overfitting through constraint mechanisms, and is well‐suited for processing high‐dimensional biological data (Liu et al. 2018). The essence of model training is to utilize a large number of samples with known chronological age y and identify an optimal set of weights β via optimization algorithms, so that the overall error between predicted age ŷ and actual age y is minimized. The most commonly used loss function is the mean squared error (see Figure 2). These models built predictive frameworks by selecting biomarkers strongly linked to chronological age, such as DNA methylation sites and blood biochemical indicators. Their main goal was to estimate biological age while focusing on stability and interpretability.

A notable model is Horvath's multi‐tissue DNA methylation clock, introduced in 2013. Based on 353 CpG sites and trained with elastic net regression across various tissues, the model achieved high‐accuracy age predictions across populations (Horvath 2013). This model was later commercialized as the DNAge Clock and has been widely used in biological age prediction (Monseur et al. 2020). Similarly, Hannum developed a blood‐specific methylation clock based on 71 CpG sites, further highlighting the effects of sex, body weight, and other factors on age prediction (Hannum et al. 2013). This indicates that the estimation of biological age relies not only on molecular markers but is also influenced by physiological and environmental factors.

To establish a connection between biological age and actual health risks, researchers introduced the concept of biological age acceleration as a transient state or as a more stable trait. Most age‐acceleration metrics are defined as residuals between predicted biological age and chronological age, and thus primarily reflect the cumulative burden of aging‐related change (Horvath 2013; Levine et al. 2018; Lu, Quach, et al. 2019; Zhang et al. 2019; Monseur et al. 2020; Xia et al. 2020; Monasso et al. 2021; Lu, Brbić, et al. 2023). These state‐like measures are informative for long‐term risk stratification but may be relatively insensitive to short‐term interventions. By contrast, trait‐like measures aim to capture the pace of aging itself. DunedinPACE (Belsky et al. 2022) is a notable example, as it quantifies the current rate of physiological decline rather than accumulated aging burden. This distinction may explain why caloric restriction (CR) reduced DunedinPACE by 2%–3%, whereas PhenoAge (Levine et al. 2018) and GrimAge (Lu, Quach, et al. 2019) showed no significant change. Further refinements have improved interpretation of residual‐based measures. Quach et al. (2017) used residuals from the Horvath clock, adjusted for cell composition, to represent intrinsic epigenetic age acceleration (IEAA), capturing cell‐intrinsic aging rates. In contrast, residuals from the Hannum clock, which include cell composition, were used to define extrinsic epigenetic age acceleration (EEAA) (Thrush et al. 2022), reflecting immune system aging. These methods deepen our understanding of how different aspects of biological aging influence health trajectories.

Nonetheless, biological aging trajectories can be nonlinear, with periods of speeding up or slowing down, which cannot be adequately modeled with linear assumptions (Peters et al. 2015). Traditional statistical models, despite their straightforward structure, have apparent limitations. First, they fail to model the nonlinear dynamics of aging. Linear models cannot capture nonlinear methylation change patterns throughout the lifespan. In contrast, nonparametric models like Gaussian Process Regression (GPR) can directly model nonlinear relationships with multidimensional function distributions (Varshavsky et al. 2023). Varshavsky et al. (2023) applied elastic net regression and ensemble machine learning to the same CpG sites for blood methylation–based age prediction, finding that elastic net produced a mean absolute error (MAE) of 2.5 years, significantly higher than the ensemble model's MAE of 1.89 years. Methods such as GPR (Varshavsky et al. 2023), support vector machines (SVMs) (Mutz et al. 2024), and tree‐based models (Chen et al. 2022) utilize flexible function fitting or feature interactions to capture dynamic inflection points in aging trajectories better. Second, many models are limited to a single dimension of data, with predictive accuracy constrained by the number of biomarkers and how well the training datasets represent the population. Montesanto developed a ridge regression model using only eight CpG sites (Montesanto et al. 2020). Its prediction error increased significantly in individuals over 90 years old (MAE > 10 years), reflecting both the underrepresentation of the oldest‐old in training sets and the increased nonlinearity of aging processes at ancient ages. Despite these limitations, linear models provided the basic foundation for biological age prediction and demonstrated strong links between biological age, mortality, and disease risk.

### 3.2 The Rise of Machine Learning: Multi‐Dimensional Integration and Breakthroughs in Predictive Accuracy (2019–2021)

With advancements in computational power and the accumulation of large‐scale datasets, the primary breakthroughs at this stage involve the ability to model nonlinear dynamics and to integrate multimodal data. Two main paradigms have emerged: ensemble learning and deep learning, each demonstrating clear advantages in high‐dimensional feature integration and complex pattern recognition, respectively. Ensemble learning combines multiple weak learners to perform predictive tasks. Standard methods include Bagging (such as Random Forests as weak learners), Boosting (like AdaBoost or Gradient Boosting), and Stacking (e.g., using Elastic Net as the meta‐model). Additionally, deep learning architectures such as convolutional neural networks (CNNs) and deep neural networks (DNNs) have greatly enhanced prediction accuracy (see Figure 2A). DNA methylation clocks, the earliest and most widely used biological age predictors, were among the first to adopt ML methods as alternatives to traditional linear models. A notable example is DeepMAge, developed by Galkin et al. (2021) which was the first deep learning–based methylation clock. Using a DNN to analyze 4930 blood methylation profiles, DeepMAge achieved an MAE of 2.77 years in an independent validation set of 1293 samples. Building on this, AltumAge, developed with a similar architecture, further reduced the MAE to 2.153 years, though its associations with health outcomes remain unclear (Shi et al. 2023). Similarly, the ResnetAge model, based on a ResNet framework, extended its applicability to multi‐tissue samples, including blood, saliva, and buccal tissues. Beyond DNA methylation, machine learning has advanced the development of aging clocks across various omics layers. A 2024 study published in Nature Medicine created a proteomic aging clock using LightGBM, combining 2897 plasma proteins from the UK Biobank and other multi‐cohort datasets. This model not only predicted all‐cause mortality but also identified 204 key protein modules associated with aging processes. Additionally, it introduced ProtAgeGap as a new measure of age acceleration, setting a fresh standard for disease risk assessment (Argentieri et al. 2024). In the same year, another study developed a cfDNA‐based aging clock by analyzing nucleosome spacing patterns. By applying machine learning to decode age‐related changes in nucleosome repeat length, the model enabled noninvasive monitoring of aging (Shtumpf et al. 2024).

Furthermore, in the context of nonlinear age prediction, the rate of aging is not constant throughout the life course, and different aging clock models cover varying predictive time spans (Porter et al. 2021; Mutz et al. 2024). Clinical monitoring of multi‐organ aging clocks yields higher‐resolution insights into biological aging (Figure 2B). Specifically, the highly dynamic nature of early life, characterized by rapid growth and biological change during gestational and minor ages, often leads to higher prediction MAE. The highest predictive accuracy, achieved by clocks like Age‐Net during the physiologically stable young to middle adulthood (Armanious et al. 2021), gives way to increased uncertainty in old age as organ decline accelerates and individual variability grows. Within this framework, the value of age‐specific aging clocks becomes evident. For the gestational period, the Placental Clock (Lee et al. 2019) and NEOAge (Graw et al. 2021) offer precise assessments of developmental processes at the inception of life. For minors (0–17 years), in addition to the PedBE Clock (McEwen et al. 2020), models such as the Childhood SL PCA Clock (Khodasevich et al. 2025) and BrainAge (Hu et al. 2023) collectively cover this developmental stage, providing multidimensional evaluation perspectives. Young adults (18–25 years) can be characterized by models including CheekAge (Shokhirev et al. 2024), AcidAGE (Kobelyatskaya, Isaev, et al. 2024), and Age‐Net (Armanious et al. 2021), which capture aging traits from epigenetic and phenotypic viewpoints. The assessment toolkit for middle‐aged adults (26–59 years) is particularly diverse, encompassing DunedinPACE (Belsky et al. 2022), XGBAge (Shim et al. 2024), as well as CheekAge (Shokhirev et al. 2024), AcidAGE (Kobelyatskaya, Isaev, et al. 2024), Age‐Net (Armanious et al. 2021), and ECG‐BA (Liu, Kuo, et al. 2025), which evaluate aging across metabolic, biochemical, and physiological dimensions. For young‐old (60–74 years) and elderly‐old adults (75–89 years), refined versions of established methylation clocks such as GrimAge2 (Lu et al. 2022) and PCHorvath (Chen et al. 2022) demonstrate robust predictive performance, while TWBAge (Huang, Pan, et al. 2025) provides organ‐specific aging information; models like fBioAge (Sternäng et al. 2019) and FaceAge (Bontempi et al. 2025) are also applicable across these two stages. In the longevity elderly (≥ 90 years), alongside Horvath‐based and Centenarian Clocks, tools such as iAge and GP‐age yield unique insights into the biology of extreme longevity. The Horvath clock, as a foundational epigenetic tool, spans from newborns to centenarians, reflecting its universal design principle. Emerging multimodal clocks such as the Microbiome Clock (Ratiner et al. 2022) and Precious1GPT (Urban et al. 2023) also show similar potential, paving the way for continuous, non‐invasive monitoring of the aging process. This lifespan perspective is further summarized, which maps biological aging velocity against the corresponding clock toolkit (see Figure 2C). Gestational age exhibits the steepest trajectory and the largest epigenetic‐age deviation. Minors' age retains rapid maturation and high prediction error, now complemented by neuroimaging and functional biomarkers. Young to middle adults' age shows plateaued change and minimal divergence, enabling low MAE prediction via epigenetic, transcriptomic, and imaging‐based clocks. Old adults' age accelerates deterioration and increases prediction error, necessitating proteomic, microbiomic, and mobility‐augmented models. Collectively, these findings establish that the aging rate is nonlinear and clock accuracy is stage‐dependent, mandating temporally resolved, multi‐omic models as the theoretical foundation for precision geroscience.

Nevertheless, machine learning–based aging clocks still face critical challenges. The complex design of deep learning models often results in limited interpretability, making it hard to identify the specific biological mechanisms behind aging. Holzscheck et al. (2021) created a neural network–based age clock that achieved high predictive accuracy using gene expression data from skin tissue. However, understanding how its internal pathway activation states relate to aging‐related pathways required manual prior knowledge, instead of being automatically discovered by the model. To address this, researchers have introduced interpretability tools such as SHapley Additive exPlanations (SHAP) (Shim et al. 2024), pathway‐constrained architectures (Johnson et al. 2020), and saliency mapping (Raghu et al. 2021), which connect black‐box predictions to biologically meaningful elements like genes and anatomical structures. Another challenge is that model performance heavily depends on the size, quality, and homogeneity of the training data. Small or inconsistent datasets can lead to overfitting and poor generalization. In cross‐population validation of the gut microbiome aging clock, dietary differences between vegetarians and non‐vegetarians altered microbial composition. They raised prediction errors, with mean squared error (MSE) increasing from 5.91 to 7.2 years (Han et al. 2025). Similarly, blood biochemistry–based clocks trained on millions of samples showed significant prediction bias when used in resource‐limited settings, where incomplete biomarker panels or batch effects reduced accuracy (Chen et al. 2022). As research advances from methylation, including genomics, proteomics, imaging, and clinical indicators, additional challenges emerge. Different modalities vary in scale, noise levels, and missing data patterns, making their direct combination more difficult. In cross‐tissue proteomic clock studies, protein concentrations in plasma versus cerebrospinal fluid can differ by orders of magnitude, which can cause models to overweight highly expressed proteins if they are not properly normalized (Johnson et al. 2020). These challenges highlight that the future of aging clock research will depend on advanced multimodal fusion techniques and mechanistic interpretability, allowing for more precise predictions and deeper biological understanding of aging processes.

### 3.3 GPT and the Transformer Revolution: Multimodal Fusion and Mechanism Discovery (2023–Present)

Before the advent of the Transformer architecture, sequence models such as recurrent neural networks (RNNs) and long short‐term memories (LSTMs) were dominant in natural language processing tasks. However, these models were difficult to train in parallel and also struggled with modeling long‐range dependencies. The Transformer encoder employs a multi‐head self‐attention mechanism to process input sequences, enabling each element's final representation to incorporate contextual information from all other elements in the sequence. This results in a set of high‐order vector representations rich in contextual cues (Vaswani et al. 2017) (see Figure 2). Generative Pre‐trained Transformer (GPT) fully harnessed the capabilities of the Transformer decoder. It fundamentally reshaped the trajectory of natural language processing and the broader AI field through innovative learning frameworks including pre‐training with fine‐tuning and later, pre‐training enhanced by human feedback reinforcement learning.

The Transformer architecture, renowned for its exceptional ability to model complex dependencies in sequential data, has been naturally adopted in bioinformatics. This makes it well‐suited for analyzing multi‐omics biological data, which is characterized by high dimensionality, complexity, and inherent dependencies often structured as sequences or graphs. For instance, one milestone achievement is AlphaFold2 (Jumper et al. 2021). This end‐to‐end deep learning model, built on the Transformer‐based Evoformer architecture, integrates and reasons with information to accurately predict the three‐dimensional structure of proteins from amino acid sequences alone, achieving atomic‐level precision. In terms of functionality, the introduction of Transformer architectures and GPTs has propelled biological age prediction toward greater interpretability and causal understanding. In 2023, Insilico Medicine launched the first Transformer‐based multimodal aging clock model, Precious1GPT, which used transfer learning to modify an age‐prediction model for disease classification. In this approach, researchers first trained a deep learning regressor on multimodal data to predict chronological age. Then, all model weights except for the final layer were frozen, and the model was fine‐tuned as a classifier to differentiate case versus control samples. To ensure model compatibility, data preprocessing steps included batch effect correction with ComBat and quantile normalization (qnorm). Model interpretability was improved using SHAP, which provided rankings of feature importance. These ranked features were crucial for both age prediction and disease‐association analysis. Using the PandaOmics platform, the researchers further examined these features to identify potential therapeutic targets. This framework not only enhanced the model's ability to handle multi‐modal data but also created a powerful tool for target discovery, bridging the gap between aging clock predictions and actionable clinical insights (Urban et al. 2023).

### 3.4 Cross‐Scale Temporal Architecture and Baseline Drift in Multi‐Omics Aging Clocks

Multi‐omics aging signals do not lie on a single temporal axis, but instead display pronounced temporal heterogeneity. Different omics layers capture biological processes unfolding over distinct timescales: DNA methylation tends to reflect longer‐term cumulative regulation and therefore behaves more like a relatively stable slow variable, whereas the transcriptome, cytokines, and parts of the metabolome are more responsive to acute perturbation and short‐term physiological fluctuation, and thus resemble fast variables (Byun et al. 2012; Furukawa et al. 2016; Chen et al. 2018). Aging itself is likewise not a linear process, but is marked by stage‐specific molecular crests and inflection points across the life course (Lehallier et al. 2019; Shen et al. 2024). In addition, aging is heterogeneous within individuals, between individuals, and across organs, such that different people, and even different tissues within the same person, may follow partially uncoupled aging trajectories (Oh et al. 2023).

Against this backdrop, the central challenge for multi‐omics aging clocks is no longer simply to increase feature number or to mechanically concatenate heterogeneous datasets, but to develop modeling strategies that explicitly accommodate distinct timescales, non‐linear transitions and biological heterogeneity. One approach is to integrate signals that encode different temporal depths. For example, gtAge combines the IgG N‐glycome, which is more reflective of long‐term cumulative biology, with the more dynamically responsive transcriptome, and uses the deep reinforcement‐learning framework AlphaSnake to learn an optimal cross‐omics feature set. Its superior performance relative to single‐omics models suggests that the joint modeling of slow and fast variables can provide a more complete approximation of biological age. A complementary strategy is to exploit the fact that different clocks are differentially sensitive to interventions and temporal perturbations. EnsembleAge, for instance, does not simply average multiple methylation clocks, but re‐integrates their outputs using the MethylGauge benchmark, which incorporates a broad spectrum of pro‐aging and rejuvenating perturbations, thereby improving the detection of intervention direction and changes in aging rate (Haghani et al. 2026). At the same time, the work of Shen et al. (2024) indicates that trajectory clustering and sliding‐window analyses, which can resolve age‐related peaks and phase transitions, are better suited than linear regression to capturing the true dynamics of aging. Organ‐specific proteomic clocks extend this logic further by showing that tissues and systems do not age synchronously; deviations in the apparent age of the heart, brain and vasculature, for example, are associated with corresponding disease risk (Oh et al. 2023). Together, these studies suggest that next‐generation aging clocks should no longer assume a single organism‐wide timescale, but instead represent long‐term accumulation, short‐term fluctuation, life‐course transitions and organ‐specific asynchrony within a unified framework. In this sense, the key to handling temporal heterogeneity is not to stack multi‐omics features into a black‐box predictor, but to build dynamic models that distinguish slow from fast variables, capture non‐linear phase changes and accommodate asynchronous aging across individuals and organs.

Baseline drift across cohorts adds a further layer of complexity to this problem, and domain adaptation or transfer learning offers a plausible way forward. The core idea is to train a model in a source domain and then adapt it to the data distribution of a target domain, thereby improving performance in new populations. Beyond approaches such as domain‐adversarial networks, which seek to reduce cohort effects by learning domain‐invariant features, generative multi‐omics frameworks represent a more ambitious direction. A representative example is AURORA, which uses generative deep learning to map seven cross‐scale biological modalities onto a shared low‐dimensional manifold, and aligns cross‐modal representations through KL‐divergence constraints and adversarial objectives. At the same time, it seeks to disentangle age‐related signals from nuisance variation, including batch effects, thereby enabling the generation of high‐quality virtual paired data. Emerging results suggest that such data‐level alignment strategies could improve the robustness of aging clocks across centres and populations and may help mitigate baseline drift.

## 4 Discussion

In summary, aging clock research has progressed from theoretical models based on single biomarkers to high‐dimensional systems enabled by multi‐omics technologies and artificial intelligence, substantially broadening the scope of biological age assessment. Compared with single‐omics clocks, the main strength of multi‐omics aging clocks is their ability to integrate complementary information across molecular layers, thereby capturing the biological heterogeneity of aging more comprehensively and, in some studies, improving age prediction and risk stratification. Their value, however, extends beyond predictive performance alone, as they also offer a richer framework for characterizing functional decline, organ‐specific aging and underlying biological mechanisms. Despite these advances, several important challenges remain, including an incomplete understanding of what aging clocks biologically measure, mismatched timescales across omics layers, baseline drift between cohorts and insufficient external validation, all of which limit clinical translation and broader applicability. Accordingly, future development should move beyond simply improving prediction accuracy toward integrative frameworks that are temporally informed, mechanistically interpretable and generalizable across populations. Such models should be able to distinguish fast from slow variables, adaptive remodeling from pathological damage, and thereby improve the utility of aging clocks for risk assessment and intervention guidance. In this effort, initiatives such as China's X‐Age project under the Aging Biomarker Consortium, together with open resources including UK Biobank, GEO, ArrayExpress, CHARLS and NHANES, provide an essential foundation for multicentre validation and cross‐population comparison. Ultimately, the field should move beyond treating aging clocks as single age estimators and instead develop decomposable risk frameworks that separate chronological drift, adaptive physiological remodeling and pathological damage accumulation. Of these components, pathological damage accumulation is most likely to represent the true biological substrate of mortality risk and the most actionable target for anti‐aging interventions.

## Author Contributions

Liying Liu and Jie Yang: conceptualization. Liying Liu, Yuanyuan Lai, Chunhui Tian, Yufei Huang, and Jianheng Hao: data curation. Liying Liu, Yuanyuan Lai, and Chunhui Tian: formal analysis. Liying Liu, Jie Yang, Yuemeng Zhao, Xiaoyan Zheng, Nihong Li, and Han Yang: resources. Nihong Li and Zheng Yu: supervision. Daqian Zhou, Tianyu Wu, and Dan Chen: visualization. Liying Liu, Yuanyuan Lai, Chunhui Tian, and Yufei Huang: writing – original draft. Liying Liu, Yuanyuan Lai, Chunhui Tian, Yufei Huang, and Jie Yang: writing – review and editing.

## Funding

This work was supported by the National Natural Science Foundation of China (82505759, 82575219, 82505351), the National Funded Postdoctoral Program (GZC20252626), the Chengdu Health Commission University Joint Innovation Fund Project (WXLH202403257), the Natural Science Foundation of Sichuan Province (No. 2024NSFSC1861), and the Technology Innovation R&D Project of Chengdu Science and Technology Bureau (No. 2024‐YF05‐00521‐SN).

## Consent

The authors have nothing to report.

## Conflicts of Interest

The authors declare no conflicts of interest.

## Data Availability

All the data was shown in the article.
